# Systemic administration of analgesic buprenorphine, but not carprofen, affects cardiomyocyte contractility in rodents

**DOI:** 10.1016/j.jmccpl.2025.100482

**Published:** 2025-09-10

**Authors:** Inez Duursma, Valentijn Jansen, Nicole Zaat, Tyler J. Kirby, Jolanda van der Velden, Diederik W.D. Kuster

**Affiliations:** a Dept Physiology, Amsterdam UMC, Amsterdam, the Netherlands; b Amsterdam Cardiovascular Sciences, Amsterdam UMC, Amsterdam, the Netherlands; c Amsterdam Movement Sciences, Tissue Function and Regeneration, Amsterdam UMC, Amsterdam, the Netherlands

**Keywords:** Analgesia, Contractility, Cardiomyocyte isolation, Research animals

## Abstract

Rodents are often used in cardiac research, where they undergo a wide variety of procedures. To ensure animal welfare, the rodents are often analgesized before, during and/or after a procedure. Contractility measurements in isolated murine cardiomyocytes are an often used method to assess function; however, little is known about the effects of analgesia on this. Therefore, we investigated the effect of systemic injection of a non-steroidal anti-inflammatory drug, carprofen (*N* = 3 mice, *n* = 273 CMs; N = 3 rats, *n* = 241 CMs) and an opioid, buprenorphine (*N* = 4 mice, *n* = 326 CMs; N = 4 rats, *n* = 308 CMs) on isolated cardiomyocytes using unloaded contractility measurements. We found that buprenorphine prolongs the relaxation of cardiomyocytes, an effect confound to the first 3 h post-isolation, whereas carprofen does not affect contractility. As analgesia might influence the stress response, we assessed the influence of carprofen and buprenorphine on the β-adrenergic receptor (AR) response. The response of cardiomyocytes to both a β-AR agonist and antagonist was not affected by carprofen or buprenorphine. *In vitro* addition of the analgesics to rat cardiomyocytes (*N* = 3 rats, *n* = 197 CMs saline, *n* = 214 CMs carprofen, *n* = 211 CMs buprenorphine) revealed that the effect of buprenorphine on contractility is caused by a systemic response rather than a direct response of cardiomyocytes specifically. Collectively, our results suggest that carprofen and buprenorphine do not affect isolated cardiomyocyte contractility if measurements are performed at least 4 h post-isolation.

## Highlights

• Carprofen does not impact rodent cardiomyocyte contractility.
• Buprenorphine significantly increases relaxation time in isolated rat cardiomyocytes.
• The effect of buprenorphine wears off within 4 h post cardiomyocyte isolation.
• β-Adrenergic receptor response is not affected by carprofen and buprenorphine.

## Graphical abstract

**Figure:**
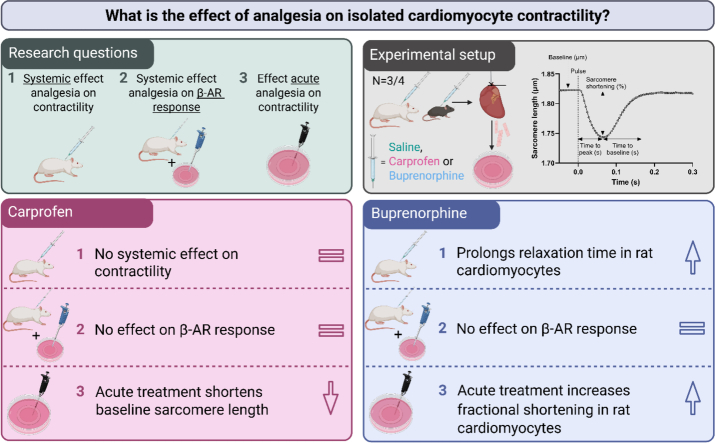

## 1 Introduction

Rodents play an essential role in research, serving as a model for studying basic human biology, disease pathogenesis and potential treatments [1]. Similarly, rodents are commonly used in cardiac research, where they undergo a wide range of complex procedures, including transverse aortic constriction or ischemia-reperfusion injury. It is widely accepted, both ethically and by regulatory standards, that animal welfare should be maximized and distress minimized [2,3]. Therefore, analgesia is often administered before, during and/or after procedures. Nonetheless, there is ongoing concern that analgesia may influence research outcomes [3,4]. Many studies involving the use of analgesia include the functional assessment of isolated cardiomyocytes [5,6], yet the impact of analgesia on the contractile mechanics and kinetics of isolated cardiomyocytes from rodents is largely unknown.

There are many different analgesics utilized in rodent research, of which the nonsteroidal anti-inflammatory drug (NSAID) carprofen and the opioid buprenorphine are the most frequently used [7]. Both analgesics desensitize the pain response through different mechanisms. NSAIDs inhibit the cyclooxygenase (COX) enzymes, which results in the inhibition of the synthesis of prostaglandins, thereby inhibiting inflammation and pain [8,9]. Carprofen is a COX-2 selective analgesic that preferably inhibits the COX-2-related pain response while avoiding COX-1 inhibition-related gastrointestinal side effects [8,10,11]. Buprenorphine binds with high affinity to the μ-, δ- and κ-opioid receptors, demonstrating partial agonism toward the first one and antagonism toward the latter two [[12], [13], [14]], resulting in antinociception. Even though buprenorphine exhibits only partial agonism toward the μ-opioid receptor, it is as effective in pain relief as full agonists of the μ-opioid receptor while having potentially lower risks of side effects such as respiratory depression and constipation [12,15,16].

Besides their ability to mitigate pain, carprofen and buprenorphine are known to affect the cardiovascular system. NSAIDs increase salt and water retention, blood pressure and afterload [[17], [18], [19]], which contribute to the increased risk of myocardial infarction and stroke and the increased risk of reoccurrence of congestive heart failure [[20], [21], [22]]. Direct exposure of cardiomyocytes to the NSAID diclofenac has been shown to affect reactive oxygen species production, calcium signaling and stress-response and cytoskeletal gene expression [[23], [24], [25]]. Whether this holds true for carprofen remains elusive. Alternatively, opioids, including buprenorphine, can lead to vasodilation, decreased heart rate and reduced fractional shortening, which might eventually lead to hypotension [26,27]. In humans, buprenorphine has been associated with a prolonged QT-interval, which predisposes to arrhythmias [28,29]. On the contrary, in a study on rats, buprenorphine has been shown to decrease QT-interval time [30]. Although some effects of both carprofen and buprenorphine on the heart have been reported, their direct effects on cardiomyocyte function are not known. This may, in turn, influence experimental outcomes.

The main goal of this study was to determine the effect of carprofen and buprenorphine on the contractility of isolated cardiomyocytes obtained from rodents. Therefore, we performed unloaded shortening measurements on primary cardiomyocytes obtained from mice or rats that were injected with carprofen, buprenorphine or saline before cardiomyocyte isolation. We show that carprofen does not affect contractile kinetics, while buprenorphine prolongs relaxation time up to 3 h after heart excision. β-AR signaling plays a crucial role in cardiac homeostasis and disease. Notably, the use of analgesia did not influence the response of cardiomyocytes to β-adrenergic stimuli. Furthermore, the addition of analgesia directly to isolated cardiomyocytes provoked a different contractile phenotype than systemic addition of analgesia. Our study shows that systemic injection of carprofen does not affect the contractility of isolated cardiomyocytes, whereas the effect of systemic buprenorphine on cardiomyocyte relaxation diminishes over time. Therefore, both analgesics can be used in rodent studies involving unloaded shortening measurements without impacting research outcomes, provided these are performed at least 4 h after heart excision.

## 2 Methods

### 2.1 Systemic analgesia and heart excision

Ten C57BL/6J mice were injected subcutaneously with either saline (0.9 % NaCl, *N* = 3), 5 mg/kg carprofen (*N* = 3) or 0.05 mg/kg buprenorphine (*N* = 4) 2 h before cardiomyocyte isolation. Saline served as a non-analgesia control. The assessor was blinded to the analgesia being injected. Similarly, 11 Wistar rats were injected subcutaneously with saline (0.9 % NaCl, *N* = 4), 5 mg/kg carprofen (*N* = 3) or 0.01 mg/kg buprenorphine (*N* = 4). Dosing regimens were based on guidelines and general practice [3]. After 2 h, the rodents were anesthetized using 5 % isoflurane. After achieving a surgical plane of anaesthesia, a large incision was made on the rodents' chest, the ribs were cut through to fully expose the heart, after which it was excised. Before cardiomyocyte isolation, the apex was cut off and flash frozen for protein analysis.

Mice and rats were all wild type and female. Mice were sacrificed between 112 and 140 days of age and rats were sacrificed between 90 and 100 days of age. Animal characteristics can be found in Tables S1 and S2. Animal handling and experiments were performed following the Guide for the Animal Care and Use Committee of the VU University Medical Center and with approval of the Guide for the Animal Care and Use Committee of the VU University Medical Center.

### 2.2 Cardiomyocyte isolation

Intact adult cardiomyocytes were isolated from mouse and rat hearts using the Langendorff set-up as previously described [31,32]. Briefly, after the heart was excised from the animal, it was washed in cold isolation Tyrode's solution (134 mM NaCl, 4 mM KCl, 10 mM HEPES, 1.2 mM MgSO_4_, 1.2 mM Na_2_HPO_4_, 11 mM glucose, pH 7.4) containing 0.2 mM EGTA (Tyrode– EGTA). Subsequently, the hearts were cannulated to a needle *via* the aorta so the heart could be perfused through the coronary vasculature with Tyrode-EGTA for 2 min at room temperature (RT). Afterwards, the hearts were perfused for 20–25 min with an enzymatic digestion solution consisting Tyrode's solution supplemented with liberase (Liberase TM Research Grade from Roche, containing highly purified Collagenase I and Collagenase II). Once the heart was digested and feeling soft, the heart was removed from the cannula and the left ventricle was separated from the right ventricle and the atria. The left ventricle was further dissected into smaller pieces and put into a series of increasingly higher calcium-containing Tyrode's solutions (0.05 mM, 0.1 mM and 0.15 mM Ca^2+^) at 37 °C. Next, the tissue pieces were agitated using a plastic Pasteur's pipet in the 0.1 mM Ca^2+^ Tyrode's solution for 3 min to create a suspension containing single cells. The suspension was filtered through a 300 μm nylon mesh strainer to filter out big clumps of cells or tissue. After 5 min, cells formed a pellet and the Tyrode's solution was replaced by cell plating medium (Medium 199, Lonza) supplemented with 1 % (*v*/v) penicillin/streptomycin and 5 % (v/v) fetal bovine serum (FBS). This cell suspension was plated on laminin-coated (10 μg/ml, Sigma-Aldrich) 35 mm dishes (MatTek) for at least 1 h at 37 °C and 5 % (*v*/v) CO_2_ to enable cells to adhere.

### 2.3 Intact cardiomyocyte contractility measurements

Cell plating medium was replaced by Tyrode's solution containing 1.0 mM calcium and 2 % (v/v) FBS 30 min before contractility measurements [31]. High-throughput contractility measurements were performed using the MultiCell system (CytoCypher BV, Amsterdam, the Netherlands) at 37 °C [33,34]. Cells were field-stimulated at 2 Hz at 15 V with a 5 ms pulse duration resulting in cardiomyocytes contracting. The sarcomere length change was recorded with a high-speed camera (250 Hz) and analyzed using IonWizard software (Ionoptix, Westwood, MA). Per experimental condition, 2–3 dishes were measured for 15 min in which as many cells as possible were measured.

#### 2.3.1 β-Adrenergic receptor modulation

To investigate the effect of analgesia on the ability of cardiomyocytes to respond to β-adrenergic receptor (AR) modulation, the β-AR pathway was either stimulated using 15 nM isoprenaline (Sigma-Aldrich) or inhibited using 5 μM propranolol (Sigma-Aldrich). For each mouse and rat that received either saline, carprofen or buprenorphine systemically, 2–3 dishes were non-treated, 2–3 dishes received isoprenaline and 2–3 dishes received propranolol. Compounds were added 5 min before measuring.

#### 2.3.2 Acute effect analgesia

The acute effect of analgesia on cardiomyocytes was directly assessed on cardiomyocytes that were retrieved from rats systemically injected with saline. Analgesia was supplemented to culture medium consisting of medium 199 (Lonza) and 2 % FBS at plasma concentration levels (saline; 20.000 ng/ml carprofen; 1 ng/ml buprenorphine) [3]. After 1 h of plating the cells, the plating medium was changed to analgesia containing culture medium for 1.5 h, after which it was exchanged for Tyrode's solution containing 1.0 mM Ca^2+^, 2 % (*v*/v) FBS, and analgesia at plasma concentration levels 30 min before the start of the measurements. Additionally, the cardiomyocytes were either non-treated or treated with isoprenaline or propranolol 5 min before the start of the measurements as explained before.

**Fig. 1:**
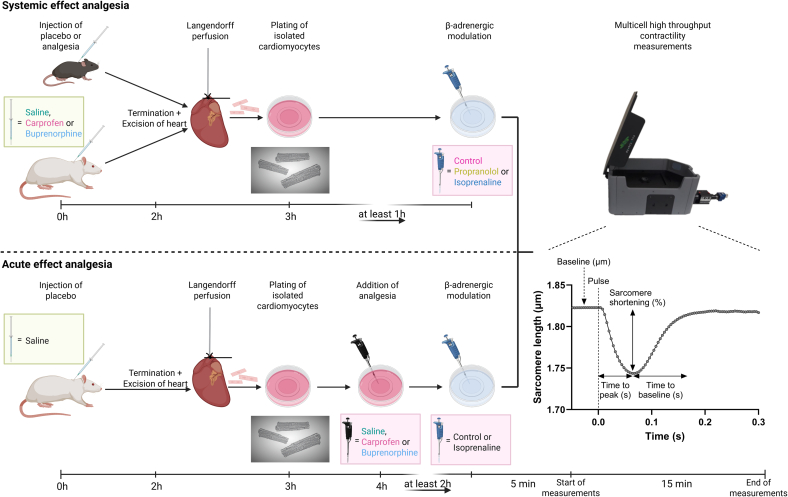
Schematic representation of method. The systemic effect of analgesia was assessed by injecting rodents subcutaneously with placebo or analgesics. Cardiomyocytes were isolated, plated, possibly treated with a β-AR modulator and measured using the multicell. The acute effect of analgesia was assessed using saline injected rats. Cardiomyocytes were isolated, plated, treated with saline or an analgesic, possibly treated with a β-AR modulator and measured using the MultiCell.

A schematic of the workflow and the different experimental conditions can be seen in Fig. 1.

### 2.4 Western blots

Homogenates of the flash frozen apex of hearts of rats injected with saline (*N* = 4), carprofen (*N* = 3) or buprenorphine (*N* = 3) were made as previously described [32]. The protein concentration of the homogenates was determined using a Pierce bicinchoninic acid (BCA) protein assay (Thermo Fisher Scientific). For each sample, 5 μg of protein was loaded on 4–15 % precast Criterion gradient gels (Bio-Rad Laboratories Inc.). Electrophoresis was performed at 100 V in sodium dodecyl sulfate (SDS) electrophoresis buffer until the dye reached the bottom of the gel (about 90 min). Wet tank transfer to a 0.45 μm PVDF Immobilon-Fl membrane (Merck) was performed at 0.3 A for 120 min. Afterwards, a Revert total protein stain (TPS, LI-COR) was performed on the membrane. Membranes were destained using a 30 % (*v*/v) methanol and 6.7 % (v/v) glacial acetic acid wash buffer and imaged using the Odyssey imager (LI-COR). Next, membranes were blocked in 5 % (*w*/*v*) milk in tris-buffered saline with 0.1 % (v/v) tween (TBS-T) for 1 h at RT. Primary antibodies were incubated overnight at 4 °C in 3 % (w/v) bovine serum albumin (BSA) in TBS-T. The next day, the membranes were washed in TBS-T after which the secondary antibodies were incubated on the membranes in 3 % (w/v) BSA in TBS-T for 1 h at RT. Membranes were washed with TBS-T, incubated with enhanced chemiluminescent (ECL) detection reagent (Amersham) and imaged using the Amersham Imager 600 (GE Healthcare Bio-Sciences AB). Protein levels were quantified using ImageQuant (Cytiva, USA) and normalized to the total protein in the sample. A list of all primary and secondary antibodies can be found in Table S3.

### 2.5 Statistical analysis

**Fig. 2:**
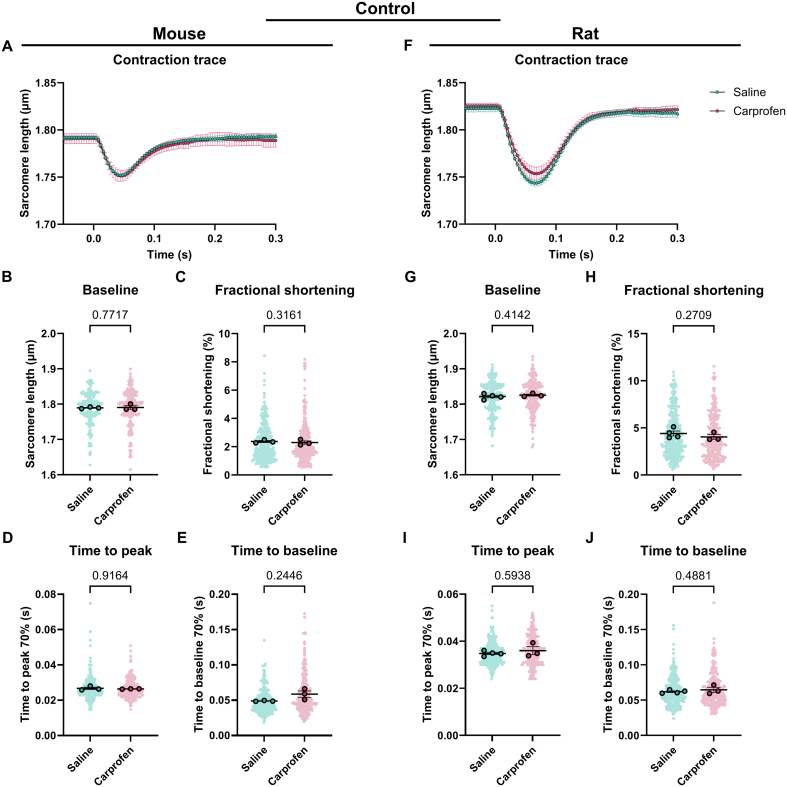
Contractility of cardiomyocytes derived from mice and rats systemically injected with saline or carprofen. (A) Average contraction traces, (B) baseline sarcomere length, (C) fractional shortening, (D) time to peak and (E) time to baseline of cardiomyocytes derived from mice injected with saline (*N* = 3, *n* = 269) or carprofen (N = 3, *n* = 273). (F) Average contraction traces, (G) baseline sarcomere length, (H) fractional shortening, (I) time to peak and (J) time to baseline of cardiomyocytes derived from rats injected with saline (*N* = 4, *n* = 314) or carprofen (*N* = 3, *n* = 241). Data are expressed as mean ± standard error of the mean. Every small symbol represents the value of a single cardiomyocyte, n, and every bigger symbol with black outline represents the average per animal, N. Linear mixed model statistics was performed, taking the analgesia as fixed effect and the different animals as random effect. Data was transformed to correct for non-normality. No post-hoc test was performed. *P*-values are shown in the graphs. Data was considered significant with *p* < 0.05.

**Fig. 3:**
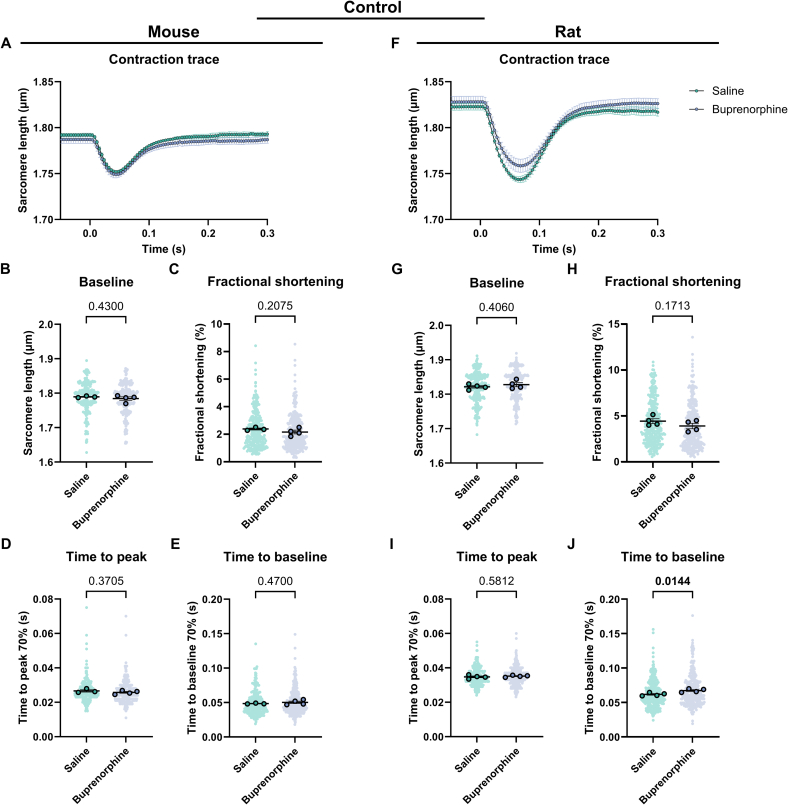
Contractility of cardiomyocytes derived from mice and rats systemically injected with saline or buprenorphine. (A) Average contraction traces, (B) baseline sarcomere length, (C) fractional shortening, (D) time to peak and (E) time to baseline of cardiomyocytes derived from mice injected with saline (N = 3, *n* = 269) or buprenorphine (*N* = 4, *n* = 326). (F) Average contraction traces, (G) baseline sarcomere length, (H) fractional shortening, (I) time to peak and (J) time to baseline of cardiomyocytes derived from rats injected with saline (N = 4, *n* = 314) or buprenorphine (N = 4, *n* = 308). Data are expressed as mean ± standard error of the mean. Every small symbol represents the value of a single cardiomyocyte, n, and every bigger symbol with black outline represents the average per animal, N. Linear mixed model statistics was performed, taking the analgesia as fixed effect and the different animals as random effect. Data was transformed to correct for non-normality. No post-hoc test was performed. *P*-values are shown in the graphs. Data was considered significant with *p* < 0.05.

**Fig. 4:**
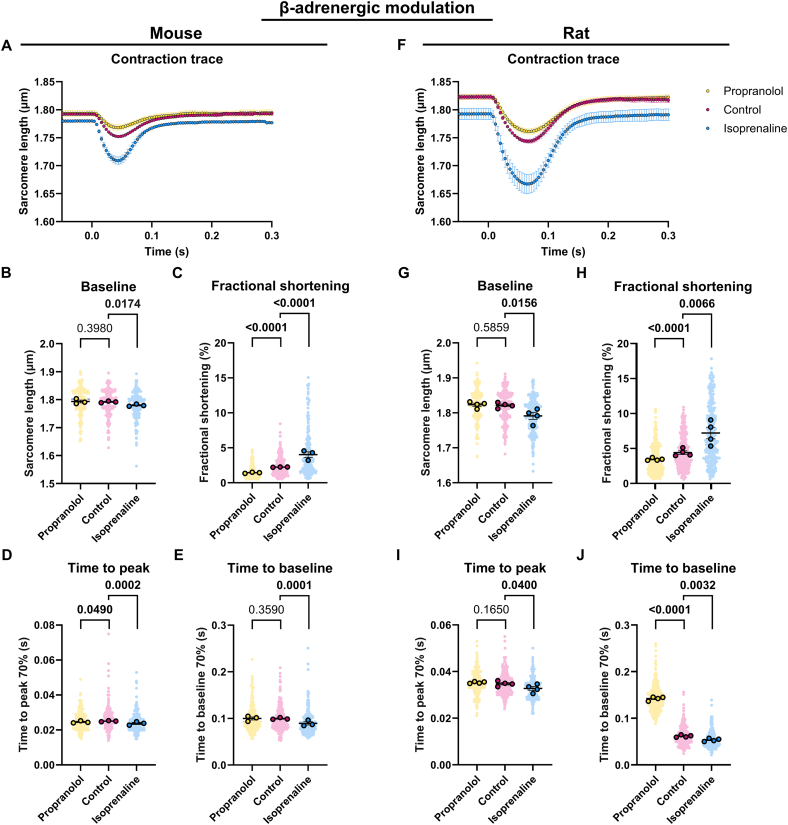
Contractility of mouse and rat cardiomyocytes treated with propranolol or isoprenaline compared to the untreated control. (A) Average contraction traces, (B) baseline sarcomere length, (C) fractional shortening, (D) time to peak and (E) time to baseline of mouse cardiomyocytes treated with propranolol (*N* = 3, *n* = 206) or isoprenaline (N = 3, *n* = 100) compared to the untreated control (N = 3, *n* = 269). (F) Average contraction traces, (G) baseline sarcomere length, (H) fractional shortening, (I) time to peak and (J) time to baseline of rat cardiomyocytes treated with propranolol (*N* = 4, *n* = 285) or isoprenaline (N = 4, *n* = 293) compared to the untreated control (N = 4, *n* = 314). Data are expressed as mean ± standard error of the mean. Every small symbol represents the value of a single cardiomyocyte, n, and every bigger symbol with black outline represents the average per animal, N. Linear mixed model statistics was performed, taking the β-adrenergic receptor modulation as fixed effect and the different animals as random effect. Data was transformed to correct for non-normality. FDR post-hoc tests were performed to correct for multiple testing. *P*-values are shown in the graphs. Data was considered significant with *p* < 0.05.

**Fig. 5:**
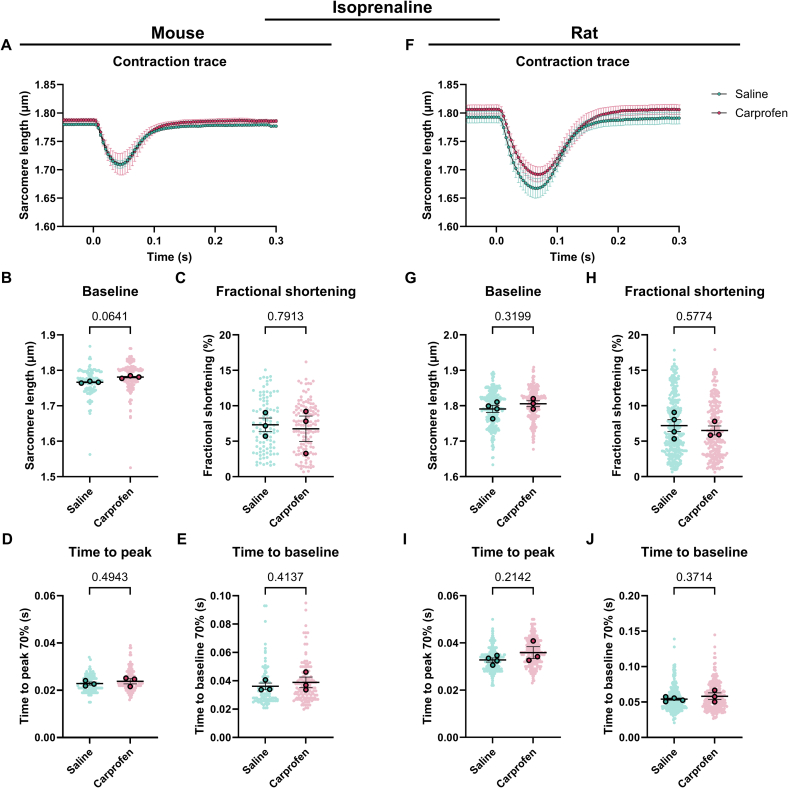
Contractility of isoprenaline-treated cardiomyocytes derived from mice and rats systemically injected with saline or carprofen. (A) Average contraction traces, (B) baseline sarcomere length, (C) fractional shortening, (D) time to peak and (E) time to baseline of cardiomyocytes treated with isoprenaline derived from mice injected with saline (*N* = 3, *n* = 84) or carprofen (N = 3, *n* = 122). (F) Average contraction traces, (G) baseline sarcomere length, (H) fractional shortening, (I) time to peak and (J) time to baseline of cardiomyocytes treated with isoprenaline derived from rats injected with saline (*N* = 4, *n* = 293) or carprofen (*N* = 3, *n* = 229). Data are expressed as mean ± standard error of the mean. Every small symbol represents the value of a single cardiomyocyte, n, and every bigger symbol with black outline represents the average per animal, N. Linear mixed model statistics was performed, taking the analgesia as fixed effect and the different animals as random effect. Data was transformed to correct for non-normality. No post-hoc test was performed. *P*-values are shown in the graphs. Data was considered significant with *p* < 0.05.

**Fig. 6:**
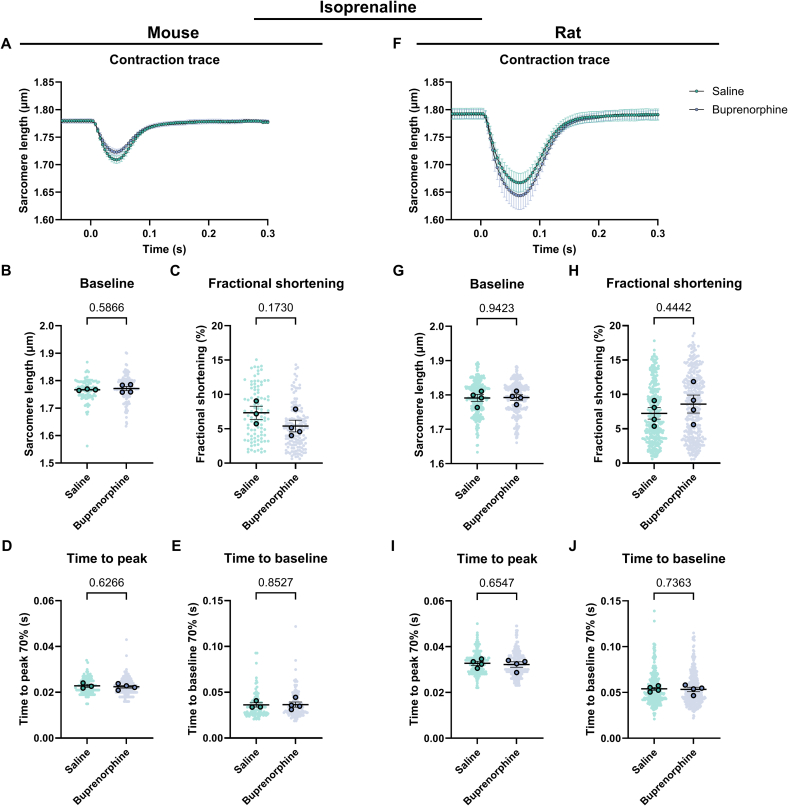
Contractility of isoprenaline-treated cardiomyocytes derived from mice and rats systemically injected with saline or buprenorphine. (A) Average contraction traces, (B) baseline sarcomere length, (C) fractional shortening, (D) time to peak and (E) time to baseline of cardiomyocytes treated with isoprenaline derived from mice injected with saline (*N* = 3, n = 84) or buprenorphine (N = 4, *n* = 131). (F) Average contraction traces, (G) baseline sarcomere length, (H) fractional shortening, (I) time to peak and (J) time to baseline of cardiomyocytes treated with isoprenaline derived from rats injected with saline (N = 4, n = 293) or buprenorphine (*N* = 3, *n* = 308). Data are expressed as mean ± standard error of the mean. Every small symbol represents the value of a single cardiomyocyte, n, and every bigger symbol with black outline represents the average per animal, N. Linear mixed model statistics was performed, taking the analgesia as fixed effect and the different animals as random effect. Data was transformed to correct for non-normality. No post-hoc test was performed. *P*-values are shown in the graphs. Data was considered significant with *p* < 0.05.

**Fig. 7:**
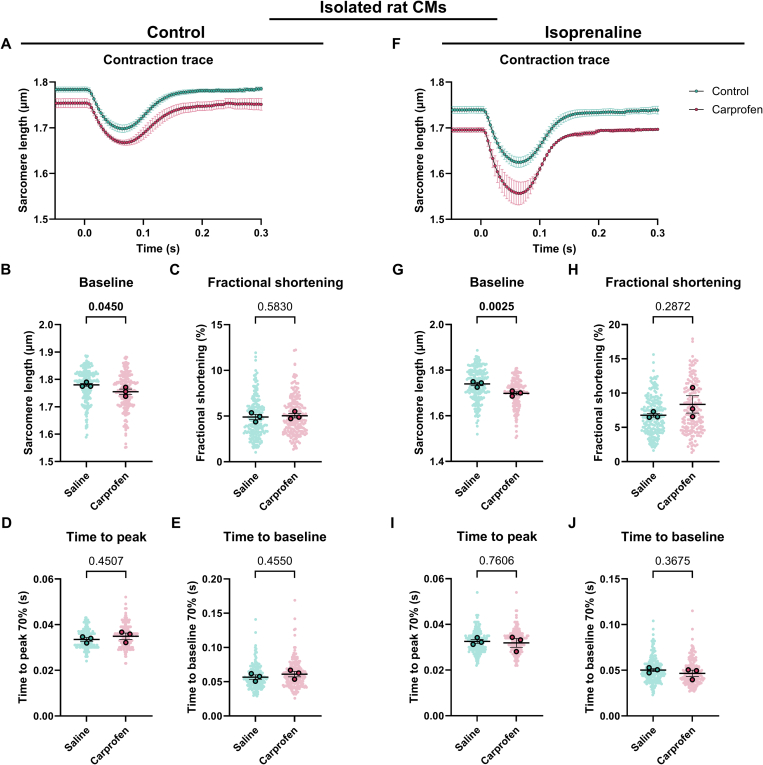
Contractility of isolated rat cardiomyocytes directly exposed to saline or carprofen in combination with no- or isoprenaline-treatment. (A) Average contraction traces, (B) baseline sarcomere length, (C) fractional shortening, (D) time to peak and (E) time to baseline of cardiomyocytes only exposed to saline (*N* = 3, *n* = 197) or carprofen (*N* = 3, *n* = 214). (F) Average contraction traces, (G) baseline sarcomere length, (H) fractional shortening, (I) time to peak and (J) time to baseline of cardiomyocytes treated with isoprenaline and saline (N = 3, *n* = 210) or carprofen (N = 3, *n* = 171). Data are expressed as mean ± standard error of the mean. Every small symbol represents the value of a single cardiomyocyte, n, and every bigger symbol with black outline represents the average per animal, N. Linear mixed model statistics was performed, taking the analgesia as fixed effect and the different animals as random effect. Data was transformed to correct for non-normality. *P*-values are shown in the graphs. Data was considered significant with *p* < 0.05.

**Fig. 8:**
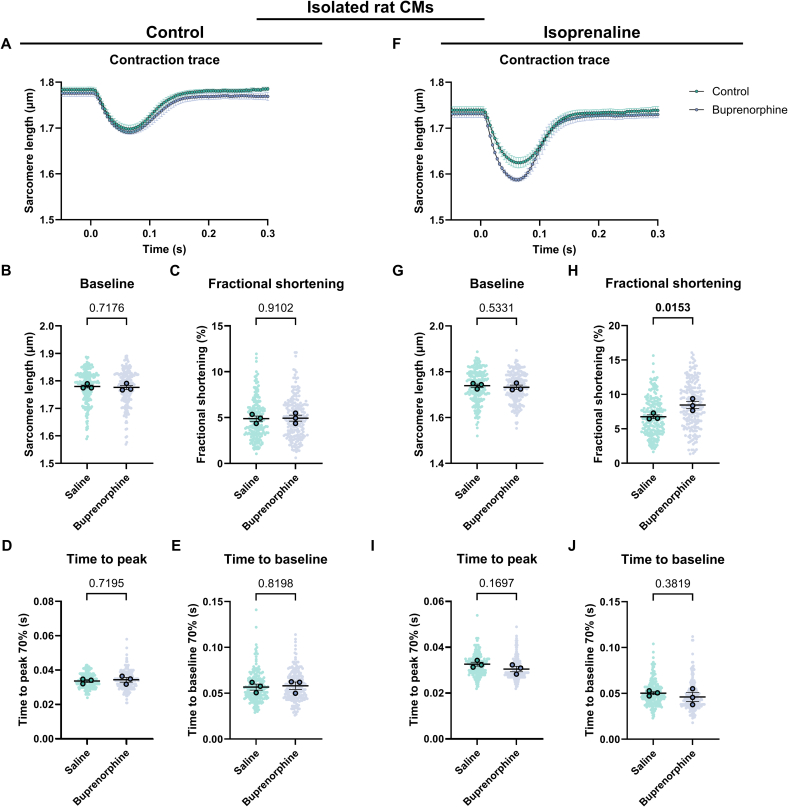
Contractility of isolated rat cardiomyocytes directly exposed to saline or buprenorphine in combination with no- or isoprenaline-treatment. (A) Average contraction traces, (B) baseline sarcomere length, (C) fractional shortening, (D) time to peak and (E) time to baseline of cardiomyocytes only exposed to saline (N = 3, n = 197) or buprenorphine (N = 3, *n* = 211). (F) Average contraction traces, (G) baseline sarcomere length, (H) fractional shortening, (I) time to peak and (J) time to baseline of cardiomyocytes treated with isoprenaline and saline (N = 3, n = 210) or buprenorphine (N = 3, n = 211). Data are expressed as mean ± standard error of the mean. Every small symbol represents the value of a single cardiomyocyte, n, and every bigger symbol with black outline represents the average per animal, N. Linear mixed model statistics was performed, taking the analgesia as fixed effect and the different animals as random effect. Data was transformed to correct for non-normality. No post-hoc test was performed. P-values are shown in the graphs. Data was considered significant with p < 0.05.

The contractility data is presented in superplots showing the mean ± standard error of the mean [35]. The statistics for the contractility data was performed in R using a linear mixed model, thereby taking the hierarchy of the data into account. The analgesia (Fig. 2, Fig. 3, Fig. 5, Fig. 6, Fig. 7, Fig. 8, S3, S4; Tables S4, S5, S12–17), β-adrenergic receptor modulation (Fig. 4; Table S10) or analgesia and time (Figs. S1, S2; Tables S6–9) were taken as the fixed effect and the different animals were taken as the random effect. Normality was assessed using a histogram of the residuals and a quantile-quantile (Q-Q) plot. Accordingly, the data was transformed to improve normality as checked by examining the histogram of the residuals and the Q-Q plot. A false discovery rate (FDR) post-hoc tests was applied to correct for multiple comparisons when more than 2 groups were compared (Fig. 4B–E, G–J). Data was considered significant with a *p* < 0.05.

The Western blot data is presented in plots showing the mean ± standard error of the mean. Due to the limited number of samples (*N* = 3/4) for true assessment of normality, non-parametric Mann-Whitney tests were performed to assess statistical significance. Data was considered significant with a *p* < 0.05.

## 3 Results

### 3.1 Systemic injection of buprenorphine, but not carprofen, affects cardiomyocyte contractility

To determine the effect of analgesics on cardiomyocytes, we assessed cardiac contractility in unloaded isolated cardiomyocytes from mice and rats. In general, the contraction of rat cardiomyocytes is greater and longer than mouse cardiomyocytes (Fig. 2A, F). The administration of carprofen systemically does not affect cardiomyocyte contractility in both mouse and rat (Fig. 2A–J, Table S4). On the other hand, the systemic injection of buprenorphine significantly prolongs the relaxation of rat cardiomyocytes indicated by the time to baseline and decreases relaxation velocity (1.1 fold, Fig. 3J, Table S5). The other parameters remain unaffected by buprenorphine (Fig. 3A–I, Table S5). In both mouse and rat cardiomyocytes, the fractional shortening seems to show a trend toward a decrease (Fig. 3C, H). Together, these data show that at baseline carprofen injection systemically does not significantly affect the contractility of unloaded isolated cardiomyocytes, while systemic injection of buprenorphine significantly prolongs the relaxation time of rat cardiomyocytes.

As the cardiomyocytes are no longer exposed to the analgesia as soon as the heart is excised, we hypothesized that perhaps the effect of the analgesia on the cardiomyocytes wears off over time. To assess the impact of time on the effect of analgesia on cardiomyocyte contractility, data were separated into 3 distinct categories (T1, T2 and T3) each representing a 45-minute interval. Independent of analgesia, we observed a significant decrease in fractional shortening over time in both mouse and rat cardiomyocytes, as well as a decrease in resting sarcomere length in rat cardiomyocytes only (Fig. S1A–D, I–J, A–D, I–J, Tables S6–9). These changes indicate progressive deterioration of the cells following isolation. In line with Fig. 2, the only significant treatment effect is the prolonged time to baseline in rat cardiomyocytes upon systemic injection with buprenorphine (Fig. S2H, L, Table S9). Interestingly, the time to baseline is significantly increased at T1 (3 h after heart excision), while it is similar to saline at T2 (3 h 45 min after heart excision) (Fig. S2L, Table S9), indicating the effect of buprenorphine wears off. In addition, the effect of systemic injection of carprofen on contractility is not significant, however, the effect of carprofen on time to peak and time to baseline in rat cardiomyocytes is dependent on time as can be observed by the significant interaction term (Fig. S1H, K, L). These results suggest that the effect of systemic injection of analgesia, specifically buprenorphine in rats, diminishes over time.

### 3.2 β-Adrenergic receptor modulation can both induce positive as negative inotropic and lusitropic effects on isolated cardiomyocytes

The guide for the Care and Use of Laboratory Animals considers pain as a stressor [36]. Stress impacts the β-AR signaling pathway through the release of catecholamines, which in turn regulate heart function by positively modulating cardiac chronotropy, inotropy and lusitropy [37]. Although increasing heart rate through β-AR stimulation can be beneficial in certain clinical conditions, such as bradycardia, long-term overstimulation of the β-AR leads to the desensitization of β-AR signaling, which results in a reduced cardiac output [38]. The latter has been observed in multiple cardiovascular diseases, such as heart failure and arrhythmias [39,40]. In these cases, it is beneficial to administer a β-AR antagonist [41,42]. To evaluate whether the response of isolated cardiomyocyte to β-AR modulation is similar to the systemic response of the whole heart, isolated cardiomyocytes from mice and rats previously injected with saline were exposed to the β-AR agonist isoprenaline or the β-AR antagonist propranolol. As expected, isoprenaline consistently reduces baseline sarcomere length, increases fractional shortening and shortens time to peak and time to baseline in both mouse and rat cardiomyocytes (Fig. 4A–J). Propranolol shows opposite effects to isoprenaline, since fractional shortening is reduced and time to baseline prolonged upon addition of propranolol (Fig. 4A, C, F, H and J, Table S10). However, the effect of propranolol compared to isoprenaline is not exactly opposite. The baseline sarcomere length in both mouse and rat cardiomyocytes, the time to baseline in mouse cardiomyocytes and the time to peak in rat cardiomyocytes remain unaffected (Fig. 4B, E, G, I and Table S11). The time to peak in mouse cardiomyocytes is shorter upon addition of propranolol (Fig. 4D). This confirms that, similar to the systemic effect, isoprenaline acts as a positive inotrope and lusitrope, while propranolol acts as a negative inotrope and lusitrope on isolated cardiomyocytes.

### 3.3 Systemic analgesics do not affect the β-adrenergic response of isolated cardiomyocytes

As explained previously, the β-AR signaling pathway is plays a crucial role in cardiac physiology and pathophysiology, and is a key target in cardiovascular therapeutics. To ensure that analgesics do not affect the β-AR response in cardiomyocytes, we exposed cardiomyocytes isolated from rodents systemically injected with analgesics to either isoprenaline or propranolol. Both carprofen and buprenorphine do not significantly influence the response of mouse and rat cardiomyocytes to isoprenaline (Fig. 5A–J, Table S12, Fig. 6A–J, Table S13). While buprenorphine prolongs the time to peak in untreated cardiomyocytes (Fig. 2J), this difference is not present upon the addition of isoprenaline (Fig. 6J). Similarly, neither analgesic significantly affected cardiomyocyte contractility of rodents exposed to propranolol (Fig. S3A–J, Table S14, Fig. S4A–J, Table S15). Collectively, systemic injection of carprofen or buprenorphine does not affect the response of isolated cardiomyocytes to β-AR modulation.

### 3.4 *In vitro* exposure of isolated cardiomyocytes to analgesics affects cardiomyocyte contractility differently than systemic exposure

Many of the described cardiac side effects of carprofen and buprenorphine are indirect, systemic effects dependent on the interplay with other organs such as the kidneys and vasculature. To further unravel if and how analgesics could affect cardiomyocyte contractility, we exposed isolated cardiomyocytes directly to carprofen or buprenorphine in the dish. Carprofen significantly reduces the baseline sarcomere length of rat cardiomyocytes at baseline and upon isoprenaline treatment (Fig. 7A, B, F, G). The other parameters are unaltered (Fig. 7C–E, H–J, Table S16). Buprenorphine increases fractional shortening and relaxation velocity in rat cardiomyocytes upon isoprenaline treatment (Fig. 8H, Table S17), while not influencing untreated cardiomyocyte contractility or baseline sarcomere length (Fig. 8A–G, I, J, Table S17). Cardiomyocytes are affected differently by carprofen and buprenorphine upon systemic or direct exposure. This suggests that the mechanism by which contractility is affected through systemic and direct exposure to analgesia differs.

In practice, analgesia will be given systemically, therefore, we tried to decipher the mechanism by which systemic injection of mainly buprenorphine influences cardiomyocyte contractility. The apex of the heart of rats injected with analgesics was used to determine protein expression. The expression of sarcomere proteins, as well as calcium handling proteins, could affect the contractility of cardiomyocytes. Carprofen and buprenorphine do not alter the expression of sarcomere proteins cardiac myosin binding protein-C (cMyBP-C) and cardiac troponin-I (cTnI) (Figs. S5A, C, S7A, C, S9, S10) and calcium handling proteins phospholamban (PLN), sarcoplasmic/endoplasmic reticulum Ca^2+^-ATPase (SERCA2) and ryanodine receptor 2 (RYR2) (Figs. S6A, D, E, S8A, D, E, S9, S10). In response to β-AR stimulation, these proteins can be phosphorylated [43]. However, carprofen and buprenorphine do not seem to influence protein kinase A (PKA)-mediated phosphorylation (Figs. S5B, D, S6B, C, F, S7B, D, S8B, C, F, S9, S10). Lastly, it is known that cytoskeletal proteins can affect contractility [[44], [45], [46]]. Nevertheless, carprofen and buprenorphine do not significantly affect α-tubulin, detyrosinated α-tubulin, acetylated α-tubulin and desmin expression (Figs. S5E–H, S7E–H, S9, S10). These results indicate that the expression of these sarcomere, calcium handling and cytoskeletal proteins cannot explain the changes in cardiomyocyte contractility due to analgesia.

## 4 Discussion

In this study, we investigated the effect of systemic injection of analgesia in rodents on the contractility of isolated cardiomyocytes. We found that buprenorphine, but not carprofen, affects the contractile kinetics of cardiomyocytes through the prolongation of relaxation time, an effect limited to the first 3 h post-isolation and which could not be explained by contractile or cytoskeletal protein expression changes. The response of cardiomyocytes to β-AR modulation was not influenced by either carprofen and buprenorphine. Direct exposure of the cardiomyocytes to the analgesics in a dish provoked different contractile changes compared to systemic injection. Taken together, this report demonstrates that systemic injection of carprofen does not affect the outcomes of high-throughput contractility measurements in isolated cardiomyocytes, whereas buprenorphine alters cardiomyocyte contractility for up to 3 h after isolation. These findings highlight the importance of considering analgesic effects in studies involving functional assessment of isolated cardiomyocytes.

Although it is known that there are multiple cardiac-specific side effects for both NSAIDs and opioids [[17], [18], [19], [20], [21], [22],[26], [27], [28], [29]], we observed relatively few side effects of carprofen and buprenorphine on cardiomyocyte contractility. Carprofen increases the risk of cardiovascular events, such as myocardial infarction and heart failure [20,47,48]. This can be mostly ascribed to the imbalance of COX enzyme products, which regulate the interaction between blood vessels and platelets, thereby increasing clotting and blood pressure [49]. Most of the cardiovascular risks of NSAIDs are linked to broader cardiovascular functions, which are removed from the equation when isolating cardiomyocytes. In addition, most side effects are related to longer-term use of NSAIDs [50]. Taken together, it is not surprising that carprofen does not affect cardiomyocyte contractility. Although there are reports that state that the β_2_-AR plays a role in the analgesic mechanism of certain NSAIDS [51], which would imply that carprofen could affect the β-AR response. Nevertheless, we did not observe this.

The main cardiovascular-related side effects of buprenorphine are slowed heart rate, decreased fractional shortening, vasodilation and prolonged QT interval [26,27]. Beyond these systemic effects, we observed a prolonged relaxation time in rat cardiomyocytes. Since we did not observe any differences in the protein expression of cardiac proteins involved in relaxation, the most plausible explanation for the buprenorphine-induced prolongation of relaxation in cardiomyocytes is an alteration in the regulation of ion channels that control sodium, calcium and potassium currents in the cell [52,53]. In accordance, it has been shown that the stimulation of opioid receptors alters the calcium current in ventricular cardiomyocytes [54,55]. Furthermore, there have been reports describing the interaction of opioids and β-ARs, which are both part of the G-protein-coupled receptors superfamily, resulting in the reduction of both L-type calcium current and contractility [56,57]. Interestingly, β-AR modulation eliminates the observed prolonged relaxation at baseline. This could indicate that there is indeed an interaction between buprenorphine and the β-ARs, which is revoked by the addition of both agonistic and antagonistic β-AR modulators at the time when the cell is no longer exposed to buprenorphine.

We observed differing responses to β-adrenergic receptor stimulation between mouse and rat cardiomyocytes. Although not statistically significant, systemic buprenorphine administration tended to blunt the increase in fractional shortening in mouse cardiomyocytes, whereas it appeared to exacerbate the increase in fractional shortening in rat cardiomyocytes. These differences in β-AR response might be due to varying β2-adrenergic receptor expression and varying sensitivity to β-adrenergic receptor stimulation between species [58]. These species-specific differences may influence the inotropic response of cardiomyocytes subjected to analgesia or other pharmacological agents in combination with β-adrenergic receptor modulation.

To investigate the mechanism by which analgesia affects cardiomyocyte contractility, cardiomyocytes were exposed to the analgesics *in vitro*. The analgesics provoked different contractile changes upon *in vitro* exposure, which suggests that the changes observed upon injecting the rodents with analgesics are likely due to systemic effects that linger after cardiomyocyte isolation rather than direct responses of the cardiomyocytes. The shorter resting sarcomere length upon *in vitro* exposure to carprofen indicates partial activation of the cardiomyocytes during diastole. A similar reduction in resting sarcomere length was observed in cardiomyocytes treated with isoprenaline. Since isoprenaline enhances PKA-mediated phosphorylation of several sarcomere proteins, such as cTnI, PLN and cMyBP-C, it might lead to the partial activation of cardiomyocytes during systole. We found out that systemic injection of carprofen does not alter sarcomere protein phosphorylation; however, it has not been checked whether *in vitro* exposure to carprofen induces PKA-mediated phosphorylation of sarcomere proteins.

*In vitro* exposure of isolated cardiomyocytes to buprenorphine increased fractional shortening, but only upon simultaneous stimulation with isoprenaline. In addition, there is no prolongation of relaxation time as observed upon systemic injection. As described previously, buprenorphine binds to the μ-, δ- and κ-opioid receptors, demonstrating partial agonism toward the first one and antagonism toward the latter two [[12], [13], [14]]. However, these receptors differ in their expression throughout the body and can also be altered upon aging and disease [[59], [60], [61]]. It has been described that only δ- and κ-opioid receptors and not μ-opioid receptors are present in isolated rat ventricular cardiomyocytes, which could explain the difference in systemic and isolated response to buprenorphine [59].

A limitation of this study was that only non-diseased rodents were used to study the effect of analgesia on contractility. It is, however, very relevant to study the contractile characteristics of rodent disease models to unravel disease mechanisms. These disease models might respond differently to systemic injection of analgesia, potentially due to different opioid receptor expression, as observed in conditions like heart failure [60]. Several rodent models, such as the homozygous *Mybpc3*_*c.2373InsG*_ HCM mouse model [62], show prolonged relaxation [63]. As buprenorphine prolongs relaxation in non-disease rodents, its use in disease models might disguise contractile differences.

Furthermore, all experiments were performed with 1.0 mM Ca^2^+ in the Tyrode's solution. This was done to increase longevity of isolated cardiomyocytes [31]. However, in a physiological environment the [Ca^2+^] is around 2 mM. The responses of pharmacological interventions are dependent on calcium concentrations inside and outside of the cell [64,65], although in our hands drug effects are more clearly seen at lower extracellular calcium concentrations (unpublished data). To be sure if the conclusion of this study holds up at normocalcaemic conditions, the experiments should be repeated in higher calcium concentrations. Additionally, it would be valuable to measure calcium transients directly. As opioid receptor stimulation can alter calcium current [54,55], the direct measurement of calcium transients can provide more information on calcium regulation in the cell than the expression levels of calcium handling proteins alone.

It would also be interesting to investigate the effect of systemic analgesia on isolated cardiomyocytes at earlier time points following isolation. In this study, the first measurements were taken 3 h post-isolation. Notably, in rats systemically injected with carprofen or buprenorphine, we observed a time-dependent effect of analgesia on cardiomyocyte contractility (Figs. S1, S2), suggesting that the initial impact of the analgesics may be underestimated. However, this does not alter the practical implications of the study, as the isolation procedure does not permit functional unloaded shortening measurements at earlier time points.

Overall, systemic injection of carprofen does not affect the contractility of isolated rodent cardiomyocytes, whereas buprenorphine prolongs the relaxation time of isolated rat cardiomyocytes. Although the exact mechanism remains unidentified, the effect of buprenorphine on contractility diminishes within 4 h after isolation. In addition, the systemic injection of carprofen and buprenorphine do not alter the contractility of both isolated mouse and rat cardiomyocytes upon β-AR modulation. Our study provides evidence that carprofen and buprenorphine can be safely used to mitigate pain in rodents utilized for mechanistic or drug discovery studies involving high-throughput contractility measurements on isolated cardiomyocytes, provided that measurements are conducted at least 4 h after cardiomyocyte isolation.

## CRediT authorship contribution statement

**Inez Duursma:** Data curation, Formal analysis, Investigation, Methodology, Visualization, Writing – original draft, Writing – review & editing. **Valentijn Jansen:** Conceptualization, Data curation, Methodology, Project administration. **Nicole Zaat:** Data curation. **Tyler J. Kirby:** Supervision, Writing – review & editing. **Jolanda van der Velden:** Supervision, Writing – review & editing. **Diederik W.D. Kuster:** Conceptualization, Supervision, Writing – review & editing.

## Funding

This work was supported by the 10.13039/501100001674Leducq Foundation (20CVD01) and the Dutch Heart Foundation (Senior Scientist Grant 03-004-2023-0102, to T.J.K).

## Declaration of competing interest

The authors declare that they have no known competing financial interests or personal relationships that could have appeared to influence the work reported in this paper.

## Data Availability

Data is available upon reasonable request. Correspondence and requests for materials or data should be addressed to DWDK.

## Appendix A Supplementary data

Supplementary material Image 1
