# Supplementary material for: Systemic administration of analgesic buprenorphine, but not carprofen, affects cardiomyocyte contractility in rodents
Source: J Mol Cell Cardiol Plus. 2025 Sep 10;14:100482. doi: 10.1016/j.jmccpl.2025.100482 (PMC12506450; doi:10.1016/j.jmccpl.2025.100482)
Supplement: Supplementary file 1 — Supplementary material [file mmc1.docx]

**Supplement**

**Table S1: Animal characteristics.**

| **Species** | **Sex** | **Strain** | **Analgesia** | **Genotype** | **Weight (g)** | **Age at termination (days)** |
| --- | --- | --- | --- | --- | --- | --- |
| Mouse | Female | C57BL/6J | Saline | Wild Type | 24.3 | 112-140 |
| Mouse | Female | C57BL/6J | Saline | Wild Type | 21.2 | 112-140 |
| Mouse | Female | C57BL/6J | Saline | Wild Type | 22.2 | 112-140 |
| Mouse | Female | C57BL/6J | Carprofen | Wild Type | 24.5 | 112-140 |
| Mouse | Female | C57BL/6J | Carprofen | Wild Type | 23.8 | 112-140 |
| Mouse | Female | C57BL/6J | Carprofen | Wild Type | 21.8 | 112-140 |
| Mouse | Female | C57BL/6J | Buprenorphine | Wild Type | 22.5 | 112-140 |
| Mouse | Female | C57BL/6J | Buprenorphine | Wild Type | 23 | 112-140 |
| Mouse | Female | C57BL/6J | Buprenorphine | Wild Type | 21.9 | 112-140 |
| Mouse | Female | C57BL/6J | Buprenorphine | Wild Type | 22.9 | 112-140 |
| Rat | Female | Wistar | Saline | Wild Type | 278 | 90 |
| Rat | Female | Wistar | Saline | Wild Type | 253 | 97 |
| Rat | Female | Wistar | Saline | Wild Type | 278 | 92 |
| Rat | Female | Wistar | Saline | Wild Type | 289 | 96 |
| Rat | Female | Wistar | Carprofen | Wild Type | 248 | 89 |
| Rat | Female | Wistar | Carprofen | Wild Type | 301 | 90 |
| Rat | Female | Wistar | Carprofen | Wild Type | 300 | 97 |
| Rat | Female | Wistar | Buprenorphine | Wild Type | 244 | 95 |
| Rat | Female | Wistar | Buprenorphine | Wild Type | 284 | 96 |
| Rat | Female | Wistar | Buprenorphine | Wild Type | 290 | 91 |
| Rat | Female | Wistar | Buprenorphine | Wild Type | 281 | 98 |

**Table S2: Comparison of characteristics of mice and rats injected with saline, carprofen or buprenorphine to show equality of groups.** Data is shown as mean ± standard deviation. N represents the number of animals. A one-way ANOVA was performed. P-values and N are shown in the table. Data was considered significant with p < 0.05.

| Mice |  | | | |
| --- | --- | --- | --- | --- |
|  | **Saline (*N*=3)** | **Carprofen (*N*=3)** | **Buprenorphine (*N*=4)** | ***P* value** |
| Weight (g) | 22.6 ± 1.6 | 23.4 ± 1.4 | 22.6 ± 0.5 | 0.64 |
| Rats |  | | | |
|  | **Saline (*N*=4)** | **Carprofen (*N*=3)** | **Buprenorphine (*N*=4)** | ***P* value** |
| Weight (g) | 274.5 ± 15.2 | 283.0 ± 30.3 | 274.8 ± 20.8 | 0.60 |
| Age at termination (days) | 93.8 ± 3.3 | 92.0 ± 4.4 | 95.0 ± 2.9 | 0.64 |

**Table S3: Overview of antibody concentrations used for western blots.**

| **Target** | **Isotype/**  **host** | **Company/product number** | **Dilution** | **Blocking reagent** |  | **Secondary antibody** | **Dilution** |
| --- | --- | --- | --- | --- | --- | --- | --- |
| cMyBP-C | Mouse | Santa Cruz SC-137180 | 1:5000 | 5% (w/v) milk |  | Goat anti-mouse IgG-HRP, Dako, P0447 | 1:5000 |
| cTnI | Rabbit | Cell signaling #4002S | 1:2500 | 5% (w/v) milk |  | Goat anti-rabbit IgG-HRP, Dako, P0448 | 1:5000 |
| PLN | Mouse | Badrilla A010-14 | 1:500 | 5% (w/v) milk |  | Goat anti-mouse IgG-HRP, Dako, P0447 | 1:5000 |
| SERCA2 | Rabbit | Cell signaling #4388 | 1:1000 | 5% (w/v) milk |  | Goat anti-rabbit IgG-HRP, Dako, P0448 | 1:5000 |
| RYR2 | Mouse | Invitrogen MA3-916 | 1:1000 | 5% (w/v) milk |  | Goat anti-mouse IgG-HRP, Dako, P0447 | 1:5000 |
| cMyBP-C p-Ser273 | Rabbit | Gift from Sadayappan lab | 1:1000 | 5% (w/v) milk |  | Goat anti-rabbit IgG-HRP, Dako, P0448 | 1:5000 |
| cTnI p-Ser23/24 | Rabbit | Cell signaling #4004 | 1:500 | 5% (w/v) milk |  | Goat anti-rabbit IgG-HRP, Dako, P0448 | 1:5000 |
| PLN p-Ser16 | Rabbit | Badrilla A010-12 | 1:500 | 5% (w/v) milk |  | Goat anti-rabbit IgG-HRP, Dako, P0448 | 1:5000 |
| PLN p-Thr17 | Rabbit | Badrilla A010-13 | 1:500 | 5% (w/v) milk |  | Goat anti-rabbit IgG-HRP, Dako, P0448 | 1:5000 |
| RYR2 p-Ser2808 | Rabbit | Badrilla A010-30AP |  | 5% (w/v) milk |  | Goat anti-rabbit IgG-HRP, Dako, P0448 | 1:5000 |
| α-tubulin | Mouse | Sigma-Aldrich T9026 | 1:10000 | 5% (w/v) milk |  | Goat anti-mouse IgG-HRP, Dako, P0447 | 1:5000 |
| Detyrosinated tubulin | Rabbit | Abcam ab48389 | 1:1000 | 5% (w/v) milk |  | Goat anti-rabbit IgG-HRP, Dako, P0448 | 1:5000 |
| Acetylated tubulin | Mouse | Sigma-Aldrich T7451 | 1:10000 | 5% (w/v) milk |  | Goat anti-mouse IgG-HRP, Dako, P0447 | 1:5000 |
| Desmin | Rabbit | Cell Signaling #5332 | 1:1000 | 5% (w/v) milk |  | Goat anti-rabbit IgG-HRP, Dako, P0448 | 1:5000 |

**Table S4: Contractility differences between untreated cardiomyocytes of mice and rats injected with saline or carprofen.** Data is shown as mean ± standard error of the mean. N represents the number of animals and n represents the number of single cardiomyocytes measured per condition. Linear mixed model statistics was performed, taking the analgesia as fixed effect and the different animals as random effect. Data was transformed to correct for non-normality. No post-hoc test were performed. P-values, N and n are shown in the table. Data was considered significant with p < 0.05. This table shows additional contractility parameters belonging to Figure 2.

| Mice | | | |
| --- | --- | --- | --- |
|  | Saline *(N=3, n=269)* | Carprofen *(N=3, n=273)* | *P* value |
| Baseline (µm) | 1.792 ± 0.0019 | 1.791 ± 0.0053 | 0.7717 |
| Fractional shortening (%) | 2.240 ± 0.0201 | 2.227 ± 0.0950 | 0.3161 |
| Time to peak 30% (s) | 0.015 ± 0.0002 | 0.015 ± 0.0003 | 0.0969 |
| Time to peak 50% (s) | 0.019 ± 0.0001 | 0.020 ± 0.0005 | 0.3847 |
| Time to peak 70% (s) | 0.025 ± 0.0002 | 0.025 ± 0.0006 | 0.9164 |
| Time to peak 90% (s) | 0.034 ± 0.0002 | 0.034 ± 0.0011 | 0.5268 |
| Departure velocity (µm/s) | 1.785 ± 0.0148 | 1.655 ± 0.1272 | 0.5878 |
| Time to baseline 30% (s) | 0.071 ± 0.0006 | 0.074 ± 0.0045 | 0.3203 |
| Time to baseline 50% (s) | 0.083 ± 0.0007 | 0.087 ± 0.0060 | 0.2744 |
| Time to baseline 70% (s) | 0.100 ± 0.0012 | 0.105 ± 0.0075 | 0.2446 |
| Time to baseline 90% (s) | 0.126 ± 0.0089 | 0.107 ± 0.0240 | 0.1056 |
| Return velocity (µm/s) | 0.832 ± 0.0083 | 1.004 ± 0.1734 | 0.2365 |
| Rats | | | |
|  | Saline *(N=4, n=314)* | Carprofen *(N=3, n=241)* | *P* value |
| Baseline (µm) | 1.823 ± 0.0037 | 1.826 ± 0.0027 | 0.4142 |
| Fractional shortening (%) | 4.379 ± 0.2664 | 3.983 ± 0.2622 | 0.2709 |
| Time to peak 30% (s) | 0.019 ± 0.0003 | 0.012 ± 0.0009 | 0.1480 |
| Time to peak 50% (s) | 0.026 ± 0.0003 | 0.026 ± 0.0012 | 0.3468 |
| Time to peak 70% (s) | 0.034 ± 0.0005 | 0.035 ± 0.0016 | 0.5938 |
| Time to peak 90% (s) | 0.048 ± 0.0009 | 0.049 ± 0.0019 | 0.6079 |
| Departure velocity (µm/s) | 2.546 ± 0.1291 | 2.226 ± 0.1945 | 0.1916 |
| Time to baseline 30% (s) | 0.102 ± 0.0027 | 0.104 ± 0.0031 | 0.4541 |
| Time to baseline 50% (s) | 0.116 ± 0.0031 | 0.118 ± 0.0039 | 0.4788 |
| Time to baseline 70% (s) | 0.132 ± 0.0030 | 0.136 ± 0.0053 | 0.4881 |
| Time to baseline 90% (s) | 0.111 ± 0.0077 | 0.113 ± 0.0128 | 0.2796 |
| Return velocity (µm/s) | 1.992 ± 0.4078 | 1.784 ± 0.5928 | 0.1341 |

**Table S5: Contractility differences between untreated cardiomyocytes of mice and rats injected with saline or buprenorphine.** Data are expressed as mean ± standard error of the mean. N represents the number of animals and n represents the number of single cardiomyocytes measured per condition. Linear mixed model statistics was performed, taking the analgesia as fixed effect and the different animals as random effect. Data was transformed to correct for non-normality. No post-hoc test were performed. P-values, N and n are shown in the table. Data was considered significant with p < 0.05. This table shows additional contractility parameters belonging to Figure 3.

| Mice | | | |
| --- | --- | --- | --- |
|  | Saline *(N=3, n=269)* | Buprenorphine *(N=4, n=326)* | *P* value |
| Baseline (µm) | 1.792 ± 0.0019 | 1.787 ± 0.0041 | 0.4300 |
| Fractional shortening (%) | 2.240 ± 0.0201 | 2.154 ± 0.0694 | 0.2075 |
| Time to peak 30% (s) | 0.015 ± 0.0002 | 0.015 ± 0.0005 | 0.4351 |
| Time to peak 50% (s) | 0.019 ± 0.0001 | 0.019 ± 0.0005 | 0.3573 |
| Time to peak 70% (s) | 0.025 ± 0.0002 | 0.025 ± 0.0006 | 0.3705 |
| Time to peak 90% (s) | 0.034 ± 0.0002 | 0.033 ± 0.0010 | 0.6803 |
| Departure velocity (µm/s) | 1.785 ± 0.0148 | 1.714 ± 0.0494 | 0.8888 |
| Time to baseline 30% (s) | 0.071 ± 0.0006 | 0.071 ± 0.0027 | 0.7408 |
| Time to baseline 50% (s) | 0.083 ± 0.0007 | 0.082 ± 0.0030 | 0.7260 |
| Time to baseline 70% (s) | 0.100 ± 0.0012 | 0.098 ± 0.0029 | 0.4700 |
| Time to baseline 90% (s) | 0.126 ± 0.0089 | 0.131 ± 0.0107 | 0.6473 |
| Return velocity (µm/s) | 0.832 ± 0.0083 | 0.824 ± 0.0696 | 0.4132 |
| Rats | | | |
|  | Saline *(N=4, n=314)* | Buprenorphine *(N=4, n=308)* | *P* value |
| Baseline (µm) | 1.823 ± 0.0037 | 1.828 ± 0.0061 | 0.4060 |
| Fractional shortening (%) | 4.379 ± 0.2664 | 3.823 ± 0.2866 | 0.1713 |
| Time to peak 30% (s) | 0.019 ± 0.0003 | 0.020 ± 0.0003 | 0.0636 |
| Time to peak 50% (s) | 0.026 ± 0.0003 | 0.027 ± 0.0004 | 0.2589 |
| Time to peak 70% (s) | 0.034 ± 0.0005 | 0.035 ± 0.0005 | 0.5812 |
| Time to peak 90% (s) | 0.048 ± 0.0009 | 0.049 ± 0.0006 | 0.8470 |
| Departure velocity (µm/s) | 2.546 ± 0.1291 | 2.175 ± 0.1534 | 0.0905 |
| Time to baseline 30% (s) | 0.102 ± 0.0027 | 0.106 ± 0.0019 | 0.2034 |
| Time to baseline 50% (s) | 0.116 ± 0.0031 | 0.120 ± 0.0022 | 0.1299 |
| Time to baseline 70% (s) | 0.132 ± 0.0030 | 0.138 ± 0.0020 | **0.0144** |
| Time to baseline 90% (s) | 0.111 ± 0.0077 | 0.107 ± 0.0128 | **0.0141** |
| Return velocity (µm/s) | 1.992 ± 0.4078 | 1.787 ± 0.2795 | **0.0131** |

**
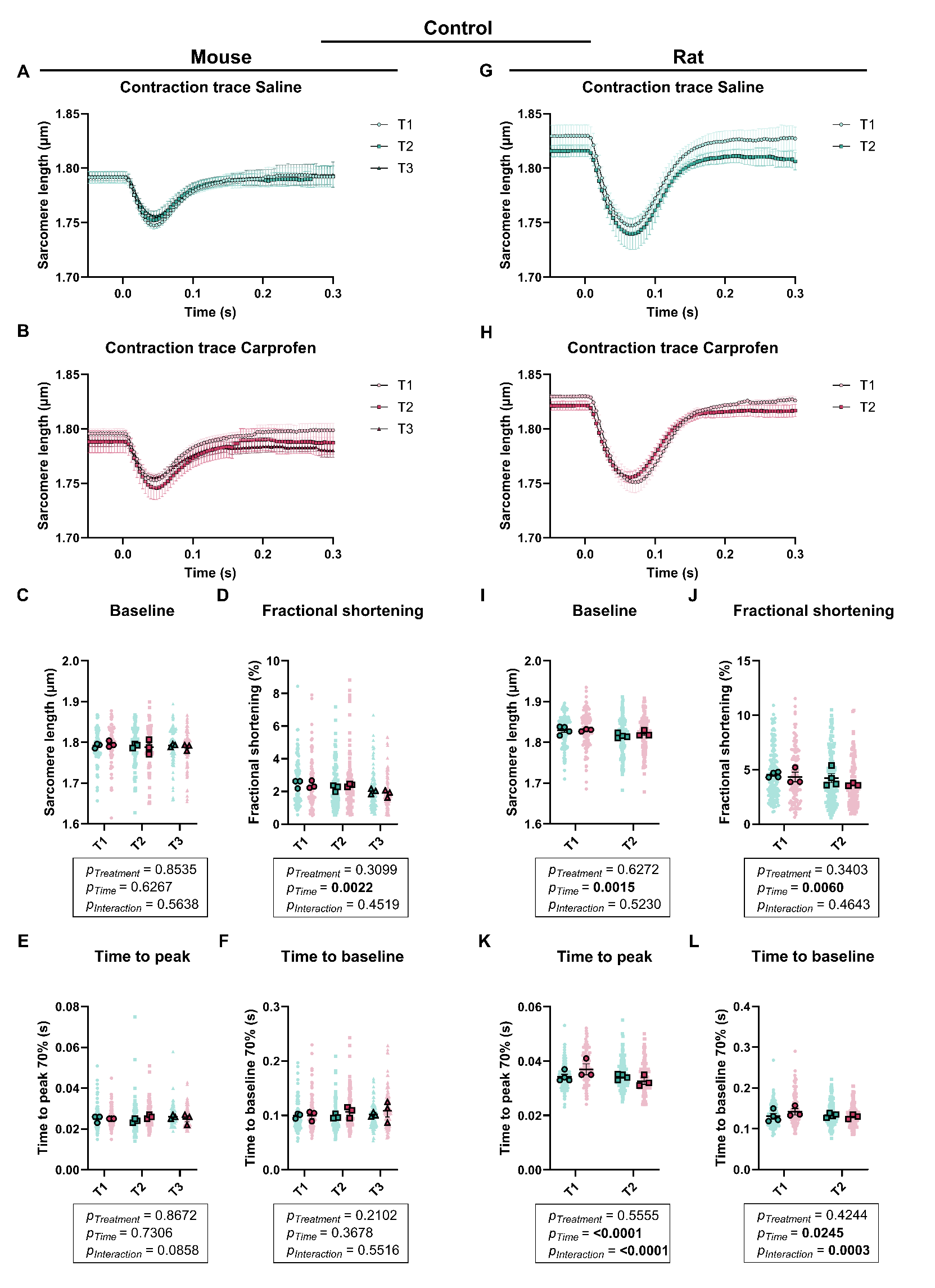
**

**Figure S1: Contractility of cardiomyocytes derived from mice and rats systemically injected with saline or carprofen over time.** (A, B) Average contraction traces, (C) baseline sarcomere length, (D) fractional shortening, (E) time to peak and (F) time to baseline at T1, T2 and T3 of cardiomyocytes derived from mice injected with saline (T1: N=3, n=99; T2: N=3, n=100; T3: N=3, n=96) or carprofen (T1: N=3, n=86; T2: N=3, n=94; T3: N=3, n=105). (G, H) Average contraction traces, (I) baseline sarcomere length, (J) fractional shortening, (K) time to peak and (L) time to baseline at T1 and T2 of cardiomyocytes derived from rats injected with saline (T1: N=4, n=157; T2: N=4, n=155) or carprofen (T1: N=3, n=123; T2: N=3, n=120). Data are expressed as mean ± standard error of the mean. Every small symbol represents the value of a single cardiomyocyte, n, and every bigger symbol with black outline represents the average per animal, N. Linear mixed model statistics was performed, taking the analgesia and time as fixed effect and the different animals as random effect. Data was transformed to correct for non-normality. A Tukey post-hoc adjustment was performed to adjust for multiple comparisons. Data was considered significant with p < 0.05. T1 = 3 hours after heart excision; T2 = 3 hours 45 minutes after heart excision; T3 = 4 hours 30 minutes after heart excision.

***
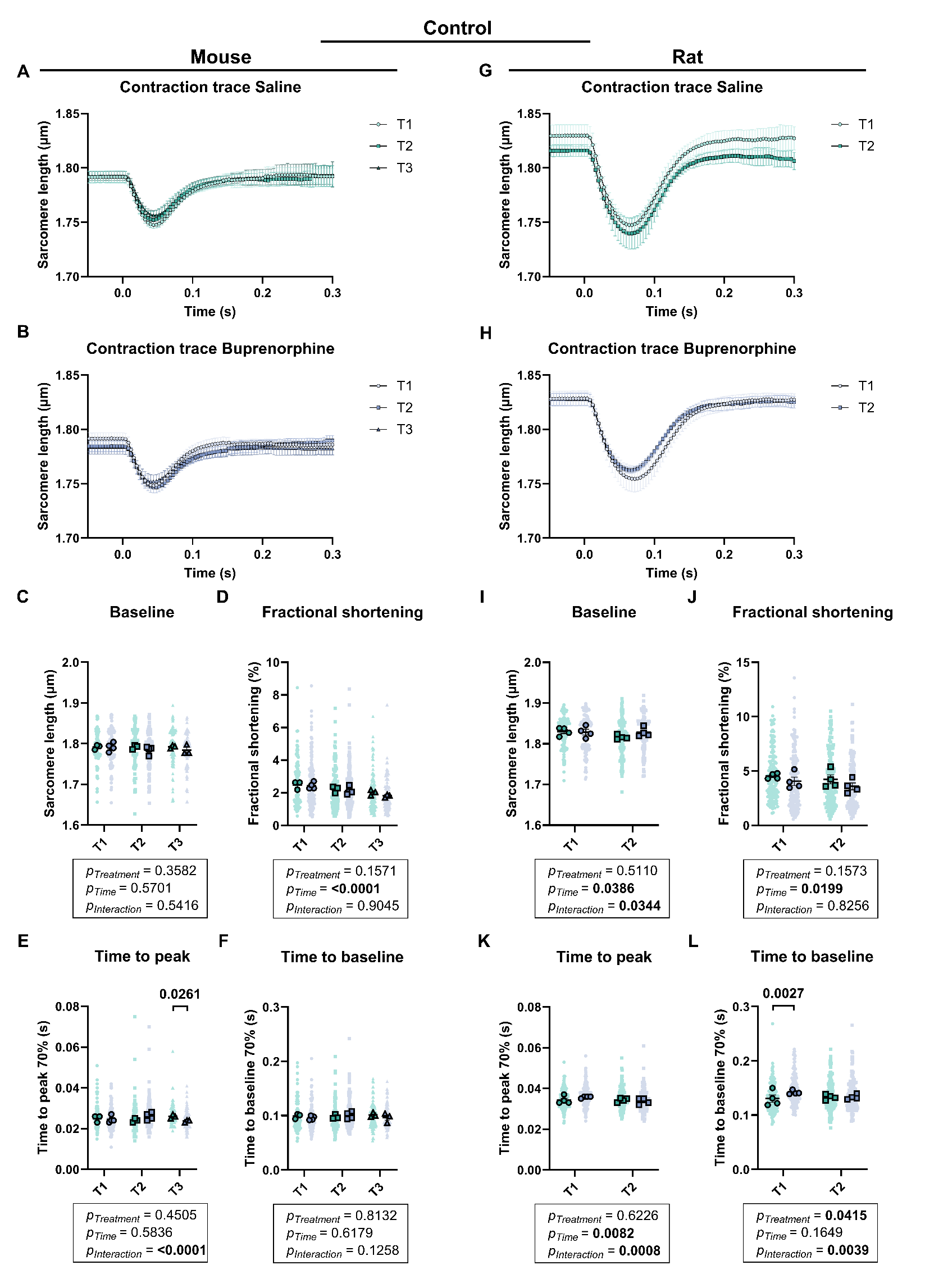
***

**Figure S2: Contractility of cardiomyocytes derived from mice and rats systemically injected with saline or buprenorphine.** (A, B) Average contraction traces, (C) baseline sarcomere length, (D) fractional shortening, (E) time to peak and (F) time to baseline of cardiomyocytes derived from mice injected with saline (T1: N=3, n=99; T2: N=3, n=100; T3: N=3, n=96) or buprenorphine (T1: N=4, n=140; T2: N=4, n=128; T3: N=4, n=91). (G, H) Average contraction traces, (I) baseline sarcomere length, (J) fractional shortening, (K) time to peak and (L) time to baseline of cardiomyocytes derived from rats injected with saline (T1: N=4, n=157; T2: N=4, n=155) or buprenorphine (T1: N=4, n=157; T2: N=4, n=157). Data are expressed as mean ± standard error of the mean. Every small symbol represents the value of a single cardiomyocyte, n, and every bigger symbol with black outline represents the average per animal, N. Linear mixed model statistics was performed, taking the analgesia and time as fixed effect and the different animals as random effect. Data was transformed to correct for non-normality. A Tukey post-hoc adjustment was performed to adjust for multiple comparisons. P-values are shown in the graphs. Data was considered significant with p < 0.05. T1 = 3 hours after heart excision; T2 = 3 hours 45 minutes after heart excision; T3 = 4 hours 30 minutes after heart excision.

**Table S6: Contractility differences between untreated cardiomyocytes of mice injected with saline or carprofen at T1, T2 and T3.** Data is shown as mean ± standard error of the mean. N represents the number of animals and n represents the number of single cardiomyocytes measured per condition. Linear mixed model statistics was performed, taking the analgesia and time as fixed effect and the different animals as random effect. Data was transformed to correct for non-normality. A Tukey post-hoc adjustment was performed to adjust for multiple comparisons. P-values, N and n are shown in the table. Data was considered significant with p < 0.05. This table shows additional contractility parameters belonging to Figure S1. T1 = 3 hours after heart excision; T2 = 3 hours 45 minutes after heart excision; T3 = 4 hours 30 minutes after heart excision.

| Mice T1 | | | |
| --- | --- | --- | --- |
|  | Saline *(N=3, n=*99*)* | Carprofen *(N=3, n=86)* | *P* value |
| Baseline (µm) | 1.792 ± 0.0033 | 1.796 ± 0.0044 | 0.5797 |
| Fractional shortening (%) | 2.482 ± 0.1492 | 2.402 ± 0.1365 | 0.1510 |
| Time to peak 30% (s) | 0.015 ± 0.0007 | 0.014 ± 0.0003 | 0.0934 |
| Time to peak 50% (s) | 0.019 ± 0.0007 | 0.019 ± 0.0001 | 0.4825 |
| Time to peak 70% (s) | 0.025 ± 0.0010 | 0.025 ± 0.0001 | 0.5439 |
| Time to peak 90% (s) | 0.034 ± 0.0013 | 0.033 ± 0.0007 | 0.6404 |
| Departure velocity (µm/s) | 1.913 ± 0.0742 | 1.775 ± 0.1633 | 0.2572 |
| Time to baseline 30% (s) | 0.070 ± 0.0032 | 0.072 ± 0.0028 | 0.6355 |
| Time to baseline 50% (s) | 0.082 ± 0.0032 | 0.083 ± 0.0045 | 0.5719 |
| Time to baseline 70% (s) | 0.099 ± 0.0027 | 0.100 ± 0.0054 | 0.3954 |
| Time to baseline 90% (s) | 0.112 ± 0.0258 | 0.117 ± 0.0286 | 0.1531 |
| Return velocity (µm/s) | 0.965 ± 0.0815 | 1.494 ± 0.4845 | 0.1499 |
| Mice T2 | | | |
|  | Saline *(N=3, n=100)* | Carprofen *(N=3, n=94)* | *P* value |
| Baseline (µm) | 1.792 ± 0.0031 | 1.788 ± 0.0101 | 0.7025 |
| Fractional shortening (%) | 2.216 ± 0.1174 | 2.392 ± 0.0553 | 0.7691 |
| Time to peak 30% (s) | 0.014 ± 0.0003 | 0.015 ± 0.0009 | 0.2279 |
| Time to peak 50% (s) | 0.019 ± 0.0003 | 0.020 ± 0.0009 | 0.0608 |
| Time to peak 70% (s) | 0.024 ± 0.006 | 0.026 ± 0.0006 | 0.0667 |
| Time to peak 90% (s) | 0.032 ± 0.0007 | 0.035 ± 0.0006 | **0.0293** |
| Departure velocity (µm/s) | 1.823 ± 0.0769 | 1.769 ± 0.0539 | 0.8853 |
| Time to baseline 30% (s) | 0.068 ± 0.0007 | 0.075 ± 0.0043 | 0.1210 |
| Time to baseline 50% (s) | 0.081 ± 0.0003 | 0.088 ± 0.0052 | 0.1091 |
| Time to baseline 70% (s) | 0.098 ± 0.0025 | 0.106 ± 0.0059 | 0.1266 |
| Time to baseline 90% (s) | 0.147 ± 0.0120 | 0.120 ± 0.0300 | 0.1699 |
| Return velocity (µm/s) | 0.833 ± 0.0936 | 0.922 ± 0.1194 | 0.5430 |
| Mice T3 | | | |
|  | Saline *(N=3, n=96)* | Carprofen *(N=3, n=105)* | *P* value |
| Baseline (µm) | 1.793 ± 0.0024 | 1.788 ± 0.0045 | 0.5406 |
| Fractional shortening (%) | 2.024 ± 0.0965 | 1.886 ± 0.1356 | 0.5802 |
| Time to peak 30% (s) | 0.016 ± 0.0003 | 0.015 ± 0.0009 | **0.0091** |
| Time to peak 50% (s) | 0.020 ± 0.0007 | 0.020 ± 0.0010 | **0.0375** |
| Time to peak 70% (s) | 0.026 ± 0.0006 | 0.025 ± 0.0015 | 0.3305 |
| Time to peak 90% (s) | 0.035 ± 0.0007 | 0.033 ± 0.0022 | 0.6701 |
| Departure velocity (µm/s) | 1.619 ± 0.645 | 1.419 ± 0.1682 | 0.4493 |
| Time to baseline 30% (s) | 0.073 ± 0.0018 | 0.075 ± 0.0068 | 0.4567 |
| Time to baseline 50% (s) | 0.085 ± 0.0017 | 0.089 ± 0.0086 | 0.3378 |
| Time to baseline 70% (s) | 0.101 ± 0.0024 | 0.108 ± 0.0112 | 0.2246 |
| Time to baseline 90% (s) | 0.118 ± 0.0310 | 0.085 ± 0.0247 | 0.4150 |
| Return velocity (µm/s) | 0.699 ± 0.0365 | 0.595 ± 0.0843 | 0.2655 |

**Table S7: Contractility differences between untreated cardiomyocytes of rats injected with saline or carprofen at T1 and T2.** Data is shown as mean ± standard error of the mean. N represents the number of animals and n represents the number of single cardiomyocytes measured per condition. Linear mixed model statistics was performed, taking the analgesia and time as fixed effect and the different animals as random effect. Data was transformed to correct for non-normality. A Tukey post-hoc adjustment was performed to adjust for multiple comparisons. P-values, N and n are shown in the table. Data was considered significant with p < 0.05. This table shows additional contractility parameters belonging to Figure S1. T1 = 3 hours after heart excision; T2 = 3 hours 45 minutes after heart excision.

| Rats T1 | | | |
| --- | --- | --- | --- |
|  | Saline *(N=4, n=157)* | Carprofen *(N=3, n=123)* | *P* value |
| Baseline (µm) | 1.830 ± 0.0051 | 1.830 ± 0.0016 | 0.9526 |
| Fractional shortening (%) | 4.531 ± 0.1289 | 4.333 ± 0.4433 | 0.5575 |
| Time to peak 30% (s) | 0.018 ± 0.0006 | 0.018 ± 0.0006 | **0.0178** |
| Time to peak 50% (s) | 0.025 ± 0.0009 | 0.025 ± 0.0009 | **0.0460** |
| Time to peak 70% (s) | 0.034 ± 0.0009 | 0.037 ± 0.0020 | 0.1019 |
| Time to peak 90% (s) | 0.046 ± 0.0015 | 0.047 ± 0.0021 | 0.1281 |
| Departure velocity (µm/s) | 2.734 ± 0.1378 | 2.744 ± 0.2545 | 0.2147 |
| Time to baseline 30% (s) | 0.099 ± 0.0041 | 0.103 ± 0.0058 | 0.0710 |
| Time to baseline 50% (s) | 0.111 ± 0.0046 | 0.116 ± 0.0046 | 0.0821 |
| Time to baseline 70% (s) | 0.131 ± 0.0070 | 0.142 ± 0.0077 | 0.0743 |
| Time to baseline 90% (s) | 0.157 ± 0.0114 | 0.138 ± 0.0254 | 0.1520 |
| Return velocity (µm/s) | 5.554 ± 3.7892 | 7.496 ± 5.9638 | 0.1771 |
| Rats T2 | | | |
|  | Saline *(N=4, n=155)* | Carprofen *(N=3, n=120)* | *P* value |
| Baseline (µm) | 1.816 ± 0.0027 | 1.821 ± 0.0042 | 0.4475 |
| Fractional shortening (%) | 4.228 ± 0.4143 | 3.634 ± 0.0815 | 0.2405 |
| Time to peak 30% (s) | 0.019 ± 0.0003 | 0.019 ± 0.007 | 0.7962 |
| Time to peak 50% (s) | 0.025 ± 0.0006 | 0.026 ± 0.0010 | 0.6241 |
| Time to peak 70% (s) | 0.034 ± 0.0005 | 0.033 ± 0.0012 | 0.4969 |
| Time to peak 90% (s) | 0.047 ± 0.0007 | 0.051 ± 0.0019 | 0.4589 |
| Departure velocity (µm/s) | 3.037 ± 0.2448 | 2.775 ± 0.2376 | 0.3098 |
| Time to baseline 30% (s) | 0.101 ± 0.0023 | 0.110 ± 0.0050 | 0.5187 |
| Time to baseline 50% (s) | 0.114 ± 0.0034 | 0.125 ± 0.0055 | 0.5279 |
| Time to baseline 70% (s) | 0.133 ± 0.0025 | 0.130 ± 0.0032 | 0.6353 |
| Time to baseline 90% (s) | 0.132 ± 0.0260 | 0.119 ± 0.0332 | 0.7653 |
| Return velocity (µm/s) | 1.829 ± 0.3859 | 1.637 ± 0.4411 | 0.1920 |

**Table S8: Contractility differences between untreated cardiomyocytes of mice injected with saline or buprenorphine at T1, T2 and T3.** Data is shown as mean ± standard error of the mean. N represents the number of animals and n represents the number of single cardiomyocytes measured per condition. Linear mixed model statistics was performed, taking the analgesia and time as fixed effect and the different animals as random effect. Data was transformed to correct for non-normality. A Tukey post-hoc adjustment was performed to adjust for multiple comparisons. P-values, N and n are shown in the table. Data was considered significant with p < 0.05. This table shows additional contractility parameters belonging to Figure S2. T1 = 3 hours after heart excision; T2 = 3 hours 45 minutes after heart excision; T3 = 4 hours 30 minutes after heart excision.

| Mice T1 | | | |
| --- | --- | --- | --- |
|  | Saline *(N=3, n=*99*)* | Buprenorphine *(N=4, n=140)* | *P* value |
| Baseline (µm) | 1.792 ± 0.0033 | 1.792 ± 0.0053 | 0.9532 |
| Fractional shortening (%) | 2.483 ± 0.1492 | 2.431 ± 0.1008 | 0.4450 |
| Time to peak 30% (s) | 0.015 ± 0.0007 | 0.014 ± 0.0006 | 0.1765 |
| Time to peak 50% (s) | 0.019 ± 0.0007 | 0.019 ± 0.0009 | 0.1369 |
| Time to peak 70% (s) | 0.025 ± 0.0010 | 0.025 ± 0.0009 | 0.2097 |
| Time to peak 90% (s) | 0.034 ± 0.0013 | 0.033 ± 0.0012 | 0.2544 |
| Departure velocity (µm/s) | 1.913 ± 0.0742 | 1.911 ± 0.0662 | 0.8935 |
| Time to baseline 30% (s) | 0.070 ± 0.0032 | 0.069 ± 0.0027 | 0.7214 |
| Time to baseline 50% (s) | 0.082 ± 0.0032 | 0.080 ± 0.0026 | 0.6746 |
| Time to baseline 70% (s) | 0.099 ± 0.0027 | 0.096 ± 0.0019 | 0.6588 |
| Time to baseline 90% (s) | 0.112 ± 0.0258 | 0.140 ± 0.0037 | 0.4761 |
| Return velocity (µm/s) | 0.965 ± 0.0815 | 0.935 ± 0.1087 | 0.5214 |
| Mice T2 | | | |
|  | Saline *(N=3, n=100)* | Buprenorphine *(N=4, n=128)* | *P* value |
| Baseline (µm) | 1.792 ± 0.0031 | 1.784 ± 0.0049 | 0.3139 |
| Fractional shortening (%) | 2.216 ± 0.1174 | 2.128 ± 0.1202 | 0.5852 |
| Time to peak 30% (s) | 0.014 ± 0.0003 | 0.015 ± 0.0008 | 0.1906 |
| Time to peak 50% (s) | 0.019 ± 0.0003 | 0.020 ± 0.0008 | 0.1370 |
| Time to peak 70% (s) | 0.024 ± 0.0006 | 0.026 ± 0.0009 | 0.0809 |
| Time to peak 90% (s) | 0.032 ± 0.0007 | 0.030 ± 0.0010 | 0.1092 |
| Departure velocity (µm/s) | 1.823 ± 0.0769 | 1.640 ± 0.0983 | 0.2064 |
| Time to baseline 30% (s) | 0.068 ± 0.0007 | 0.073 ± 0.0027 | 0.1699 |
| Time to baseline 50% (s) | 0.081 ± 0.0003 | 0.083 ± 0.0032 | 0.2014 |
| Time to baseline 70% (s) | 0.098 ± 0.0025 | 0.101 ± 0.0033 | 0.2018 |
| Time to baseline 90% (s) | 0.147 ± 0.0120 | 0.125 ± 0.0218 | 0.6160 |
| Return velocity (µm/s) | 0.833 ± 0.0936 | 0.884 ± 0.1215 | 0.7187 |
| Mice T3 | | | |
|  | Saline *(N=3, n=96)* | Buprenorphine *(N=3, n=91)* | *P* value |
| Baseline (µm) | 1.793 ± 0.0024 | 1.784 ± 0.0068 | 0.2751 |
| Fractional shortening (%) | 2.024 ± 0.0965 | 1.813 ± 0.0523 | 0.2857 |
| Time to peak 30% (s) | 0.016 ± 0.0003 | 0.014 ± 0.0001 | **0.0286** |
| Time to peak 50% (s) | 0.020 ± 0.0007 | 0.018 ± 0.0003 | **0.0196** |
| Time to peak 70% (s) | 0.026 ± 0.0006 | 0.024 ± 0.0003 | **0.0261** |
| Time to peak 90% (s) | 0.035 ± 0.0007 | 0.032 ± 0.0015 | **0.0430** |
| Departure velocity (µm/s) | 1.619 ± 0.0645 | 1.536 ± 0.0222 | 0.5868 |
| Time to baseline 30% (s) | 0.073 ± 0.0018 | 0.070 ± 0.0038 | 0.6014 |
| Time to baseline 50% (s) | 0.085 ± 0.0017 | 0.081 ± 0.0047 | 0.7854 |
| Time to baseline 70% (s) | 0.101 ± 0.0024 | 0.097 ± 0.0050 | 0.7906 |
| Time to baseline 90% (s) | 0.118 ± 0.0310 | 0.139 ± 0.0069 | 0.9681 |
| Return velocity (µm/s) | 0.699 ± 0.0365 | 0.626 ± 0.0336 | 0.1432 |

**Table S9: Contractility differences between untreated cardiomyocytes of rats injected with saline or buprenorphine at T1 and T2.** Data is shown as mean ± standard error of the mean. N represents the number of animals and n represents the number of single cardiomyocytes measured per condition. Linear mixed model statistics was performed, taking the analgesia and time as fixed effect and the different animals as random effect. Data was transformed to correct for non-normality. A Tukey post-hoc adjustment was performed to adjust for multiple comparisons. P-values, N and n are shown in the table. Data was considered significant with p < 0.05. This table shows additional contractility parameters belonging to Figure S2. T1 = 3 hours after heart excision; T2 = 3 hours 45 minutes after heart excision.

| Rats T1 | | | |
| --- | --- | --- | --- |
|  | Saline *(N=4, n=157)* | Buprenorphine *(N=4, n=157)* | *P* value |
| Baseline (µm) | 1.830 ± 0.0051 | 1.828 ± 0.0069 | 0.8418 |
| Fractional shortening (%) | 4.531 ± 0.1289 | 4.058 ± 0.3841 | 0.2047 |
| Time to peak 30% (s) | 0.019 ± 0.0007 | 0.020 ± 0.0003 | **0.0218** |
| Time to peak 50% (s) | 0.026 ± 0.0008 | 0.027 ± 0.0003 | **0.0295** |
| Time to peak 70% (s) | 0.034 ± 0.0009 | 0.036 ± 0.0002 | 0.0596 |
| Time to peak 90% (s) | 0.048 ± 0.0017 | 0.050 ± 0.0005 | 0.1494 |
| Departure velocity (µm/s) | 2.622 ± 0.0450 | 2.240 ± 0.1964 | 0.0670 |
| Time to baseline 30% (s) | 0.101 ± 0.0048 | 0.110 ± 0.0015 | **0.0449** |
| Time to baseline 50% (s) | 0.114 ± 0.0059 | 0.124 ± 0.0020 | **0.0221** |
| Time to baseline 70% (s) | 0.131 ± 0.0070 | 0.142 ± 0.0018 | **0.0027** |
| Time to baseline 90% (s) | 0.120 ± 0.0198 | 0.133 ± 0.0259 | **0.0063** |
| Return velocity (µm/s) | 2.274 ± 0.5881 | 1.357 ± 0.1533 | **0.0080** |
| Rats T2 | | | |
|  | Saline *(N=4, n=155)* | Buprenorphine *(N=4, n=157)* | *P* value |
| Baseline (µm) | 1.816 ± 0.0027 | 1.828 ± 0.0057 | 0.1733 |
| Fractional shortening (%) | 4.228 ± 0.4143 | 3.589 ± 0.3140 | 0.1545 |
| Time to peak 30% (s) | 0.019 ± 0.0003 | 0.019 ± 0.0005 | 0.4224 |
| Time to peak 50% (s) | 0.026 ± 0.0003 | 0.026 ± 0.0005 | 0.7789 |
| Time to peak 70% (s) | 0.034 ± 0.0005 | 0.034 ± 0.0008 | 0.2534 |
| Time to peak 90% (s) | 0.048 ± 0.0007 | 0.047 ± 0.0009 | 0.2577 |
| Departure velocity (µm/s) | 2.471 ± 0.2593 | 2.110 ± 0.1608 | 0.1819 |
| Time to baseline 30% (s) | 0.103 ± 0.0017 | 0.103 ± 0.0023 | 0.8820 |
| Time to baseline 50% (s) | 0.117 ± 0.0021 | 0.116 ± 0.0024 | 0.8306 |
| Time to baseline 70% (s) | 0.133 ± 0.0025 | 0.133 ± 0.0024 | 0.5514 |
| Time to baseline 90% (s) | 0.102 ± 0.0197 | 0.081 ± 0.0025 | 0.0880 |
| Return velocity (µm/s) | 1.711 ± 0.3596 | 2.218 ± 0.5644 | 0.0728 |

**Table S10: Contractility differences between propranolol-, un- or isoprenaline-treated cardiomyocytes of mice and rats systemically injected with saline.** Data are expressed as mean ± standard error of the mean. N represents the number of animals and n represents the number of single cardiomyocytes measured per condition. Every small symbol represents the value of a single cardiomyocyte, n, and every bigger symbol with black outline represents the average per animal, N. Linear mixed model statistics was performed, taking the β-adrenergic receptor modulation as fixed effect and the different animals as random effect. Data was transformed to correct for non-normality. FDR post-hoc tests were performed to correct for multiple testing. P-values, N and n are shown in the table. Data was considered significant with p < 0.05. This table shows additional contractility parameters belonging to Figure 4.

| Mice | | | | | | | |
| --- | --- | --- | --- | --- | --- | --- | --- |
|  | | Propranolol *(N=3 , n=206)* | | Control *(N=3, n=269)* | Isoprenaline *(N=3, n=100)* | *P* value | |
|  |  |  |  |  |  | Prop vs Con | Con vs Iso |
| Baseline (µm) | | 1.794 ± 0.0051 | | 1.792 ± 0.0019 | 1.780 ± 0.0023 | 0.3980 | **0.0174** |
| Fractional shortening (%) | | 1.435 ± 0.0512 | | 2.240 ± 0.0201 | 4.020 ± 0.4092 | **<0.0001** | **<0.0001** |
| Time to peak 30% (s) | | 0.015 ± 0.0004 | | 0.015 ± 0.0002 | 0.014 ± 0.0002 | 0.6950 | **0.0200** |
| Time to peak 50% (s) | | 0.020 ± 0.0004 | | 0.019 ± 0.0001 | 0.018 ± 0.0003 | 0.4303 | **0.0420** |
| Time to peak 70% (s) | | 0.025 ± 0.0004 | | 0.025 ± 0.0002 | 0.024 ± 0.0006 | **0.0490** | **0.0002** |
| Time to peak 90% (s) | | 0.032 ± 0.0005 | | 0.034 ± 0.0002 | 0.032 ± 0.0009 | 0.2567 | 0.2339 |
| Departure velocity (µm/s) | | 1.207 ± 0.0616 | | 1.785 ± 0.0148 | 3.495 ± 0.3450 | **0.0011** | **0.0004** |
| Time to baseline 30% (s) | | 0.070 ± 0.0022 | | 0.071 ± 0.0006 | 0.067 ± 0.0020 | 0.8351 | 0.3806 |
| Time to baseline 50% (s) | | 0.082 ± 0.0025 | | 0.083 ± 0.0007 | 0.076 ± 0.0026 | 0.8950 | 0.1311 |
| Time to baseline 70% (s) | | 0.100 ± 0.0032 | | 0.100 ± 0.0012 | 0.089 ± 0.0036 | 0.3590 | **<0.0001** |
| Time to baseline 90% (s) | | 0.122 ± 0.0073 | | 0.126 ± 0.0089 | 0.114 ± 0.0044 | 0.3363 | 0.1007 |
| Return velocity (µm/s) | | 0.599 ± 0.0675 | | 0.832 ± 0.0083 | 1.895 ± 0.1247 | **0.0007** | **0.0007** |
| Rats |  | |  | | | | |
|  | | Propranolol *(N=4, n=285)* | | Control *(N=4, n=314)* | Isoprenaline *(N=4, n=293)* | *P* value | |
|  |  |  |  |  |  | Prop vs Con | Con vs Iso |
| Baseline (µm) | | 1.823 ± 0.0047 | | 1.823 ± 0.0037 | 1.793 ± 0.0100 | 0.5859 | **0.0156** |
| Fractional shortening (%) | | 3.181 ± 0.2974 | | 4.379 ± 0.2664 | 7.026 ± 0.7823 | **<0.0001** | **0.0066** |
| Time to peak 30% (s) | | 0.020 ± 0.0004 | | 0.019 ± 0.0003 | 0.017 ± 0.0006 | **0.0004** | 0.1044 |
| Time to peak 50% (s) | | 0.026 ± 0.0005 | | 0.026 ± 0.0003 | 0.023 ± 0.0009 | **0.0148** | 0.0634 |
| Time to peak 70% (s) | | 0.035 ± 0.0006 | | 0.034 ± 0.0005 | 0.032 ± 0.0010 | 0.1650 | **0.0400** |
| Time to peak 90% (s) | | 0.049 ± 0.0005 | | 0.048 ± 0.0009 | 0.046 ± 0.0011 | 0.3733 | 0.3733 |
| Departure velocity (µm/s) | | 1.846 ± 0.1370 | | 2.546 ± 0.1291 | 4.394 ± 0.5630 | **<0.0001** | **0.0079** |
| Time to baseline 30% (s) | | 0.109 ± 0.0031 | | 0.102 ± 0.0027 | 0.099 ± 0.0025 | **0.0006** | 0.2486 |
| Time to baseline 50% (s) | | 0.125 ± 0.0044 | | 0.116 ± 0.0031 | 0.110 ± 0.0029 | **0.0001** | 0.0860 |
| Time to baseline 70% (s) | | 0.143 ± 0.0057 | | 0.132 ± 0.0030 | 0.124 ± 0.0037 | **<0.0001** | **0.0032** |
| Time to baseline 90% (s) | | 0.127 ± 0.0168 | | 0.111 ± 0.0077 | 0.133 ± 0.0118 | **0.0006** | **0.0339** |
| Return velocity (µm/s) | | 1.795 ± 0.6115 | | 1.992 ± 0.4078 | 3.378 ± 0.1763 | **<0. 0001** | **0.0081** |

**Table S11: Fold change of contractility parameters upon addition of propranolol or isoprenaline relative to the control in mouse and rat.**

|  | Mouse | | Rat | |
| --- | --- | --- | --- | --- |
|  | Propranolol | Isoprenaline | Propranolol | Isoprenaline |
| Time to baseline | 1.00 | 0.99 | 1.00 | 0.98 |
| Fractional shortening | 0.64 | 1.79 | 0.78 | 1.64 |
| Time to peak | 0.98 | 0.95 | 1.01 | 0.94 |
| Time to baseline | 1.00 | 0.90 | 2.3 | 0.87 |

**Table S12: Contractility differences between isoprenaline-treated cardiomyocytes of mice and rats systemically injected with saline or carprofen.** Data are expressed as mean ± standard error of the mean. N represents the number of animals and n represents the number of single cardiomyocytes measured per condition. Linear mixed model statistics was performed, taking the analgesia as fixed effect and the different animals as random effect. Data was transformed to correct for non-normality. No post-hoc test were performed. P-values, N and n are shown in the table. Data was considered significant with p < 0.05. This table shows additional contractility parameters belonging to Figure 5.

| Mice | | | |
| --- | --- | --- | --- |
|  | Saline *(N=3, n=84)* | Carprofen *(N=3, n=122)* | *P* value |
| Baseline (µm) | 1.780 ± 0.0023 | 1.787 ± 0.0033 | 0.0641 |
| Fractional shortening (%) | 4.020 ± 0.4092 | 4.393 ± 0.9168 | 0.7913 |
| Time to peak 30% (s) | 0.014 ± 0.0002 | 0.014 ± 0.0002 | 0.9966 |
| Time to peak 50% (s) | 0.018 ± 0.0003 | 0.019 ± 0.0002 | 0.5039 |
| Time to peak 70% (s) | 0.024 ± 0.0006 | 0.024 ± 0.0003 | 0.4943 |
| Time to peak 90% (s) | 0.032 ± 0.0009 | 0.033 ± 0.0008 | 0.2742 |
| Departure velocity (µm/s) | 3.495 ± 0.3450 | 3.631 ± 0.7603 | 0.6563 |
| Time to baseline 30% (s) | 0.067 ± 0.0020 | 0.070 ± 0.0037 | 0.3357 |
| Time to baseline 50% (s) | 0.076 ± 0.0026 | 0.081 ± 0.0042 | 0.2881 |
| Time to baseline 70% (s) | 0.089 ± 0.0036 | 0.095 ± 0.0052 | 0.4137 |
| Time to baseline 90% (s) | 0.114 ± 0.0044 | 0.109 ± 0.0097 | 0.4338 |
| Return velocity (µm/s) | 1.895 ± 0.1247 | 2.279 ± 0.1375 | 0.9096 |
| Rats | | | |
|  | Saline *(N=4, n=293)* | Carprofen *(N=3, n=229)* | *P* value |
| Baseline (µm) | 1.793 ± 0.0100 | 1.806 ± 0.0080 | 0.3199 |
| Fractional shortening (%) | 7.026 ± 0.7823 | 6.455 ± 0.6306 | 0.5774 |
| Time to peak 30% (s) | 0.017 ± 0.0006 | 0.020 ± 0.0009 | 0.1874 |
| Time to peak 50% (s) | 0.023 ± 0.0009 | 0.026 ± 0.0022 | 0.2107 |
| Time to peak 70% (s) | 0.032 ± 0.0010 | 0.035 ± 0.0029 | 0.2142 |
| Time to peak 90% (s) | 0.046 ± 0.0011 | 0.050 ± 0.0037 | 0.2285 |
| Departure velocity (µm/s) | 4.394 ± 0.5630 | 3.732 ± 0.6089 | 0.5272 |
| Time to baseline 30% (s) | 0.099 ± 0.0025 | 0.107 ± 0.0075 | 0.3173 |
| Time to baseline 50% (s) | 0.110 ± 0.0029 | 0.119 ± 0.0087 | 0.2983 |
| Time to baseline 70% (s) | 0.124 ± 0.0037 | 0.134 ± 0.0102 | 0.3714 |
| Time to baseline 90% (s) | 0.133 ± 0.0118 | 0.150 ± 0.0047 | 0.2495 |
| Return velocity (µm/s) | 3.378 ± 0.1763 | 3.660 ± 0.8085 | 0.5307 |

**Table S13: Contractility differences between isoprenaline-treated cardiomyocytes of mice and rats systemically injected with saline or buprenorphine.** Data are expressed as mean ± standard error of the mean. N represents the number of animals and n represents the number of single cardiomyocytes measured per condition. Linear mixed model statistics was performed, taking the analgesia as fixed effect and the different animals as random effect. Data was transformed to correct for non-normality. No post-hoc test were performed. P-values, N and n are shown in the table. Data was considered significant with p < 0.05. This table shows additional contractility parameters belonging to Figure 6.

| Mice | | | |
| --- | --- | --- | --- |
|  | Saline *(N=3, n=84)* | Buprenorphine *(N=4, n=131)* | *P* value |
| Baseline (µm) | 1.780 ± 0.0023 | 1.779 ± 0.0041 | 0.5866 |
| Fractional shortening (%) | 4.020 ± 0.4092 | 3.213 ± 0.3222 | 0.1730 |
| Time to peak 30% (s) | 0.014 ± 0.0002 | 0.014 ± 0.0006 | 0.8284 |
| Time to peak 50% (s) | 0.018 ± 0.0003 | 0.019 ± 0.0007 | 0.7553 |
| Time to peak 70% (s) | 0.024 ± 0.0006 | 0.024 ± 0.0008 | 0.6266 |
| Time to peak 90% (s) | 0.032 ± 0.0009 | 0.033 ± 0.0010 | 0.6224 |
| Departure velocity (µm/s) | 3.495 ± 0.3450 | 2.704 ± 0.3249 | 0.1958 |
| Time to baseline 30% (s) | 0.067 ± 0.0020 | 0.069 ± 0.0028 | 0.6752 |
| Time to baseline 50% (s) | 0.076 ± 0.0026 | 0.079 ± 0.0032 | 0.6573 |
| Time to baseline 70% (s) | 0.089 ± 0.0036 | 0.093 ± 0.0043 | 0.8527 |
| Time to baseline 90% (s) | 0.114 ± 0.0044 | 0.121 ± 0.0078 | 0.6369 |
| Return velocity (µm/s) | 1.895 ± 0.1247 | 1.419 ± 0.2304 | 0.2413 |
| Rats | | | |
|  | Saline *(N=4, n=293)* | Buprenorphine *(N=4, n=308)* | *P* value |
| Baseline (µm) | 1.793 ± 0.0100 | 1.792 ± 0.0082 | 0.9423 |
| Fractional shortening (%) | 7.026 ± 0.7823 | 8.341 ± 1.2027 | 0.4442 |
| Time to peak 30% (s) | 0.017 ± 0.0006 | 0.0169 ± 0.0008 | 0.7819 |
| Time to peak 50% (s) | 0.023 ± 0.0009 | 0.0233 ± 0.0009 | 0.7015 |
| Time to peak 70% (s) | 0.032 ± 0.0010 | 0.0314 ± 0.0014 | 0.6547 |
| Time to peak 90% (s) | 0.046 ± 0.0011 | 0.0458 ± 0.0014 | 0.6835 |
| Departure velocity (µm/s) | 4.394 ± 0.5630 | 5.472 ± 0.9451 | 0.3821 |
| Time to baseline 30% (s) | 0.099 ± 0.0025 | 0.1014 ± 0.0031 | 0.7618 |
| Time to baseline 50% (s) | 0.110 ± 0.0029 | 0.1126 ± 0.0032 | 0.8772 |
| Time to baseline 70% (s) | 0.124 ± 0.0037 | 0.1263 ± 0.0035 | 0.7363 |
| Time to baseline 90% (s) | 0.133 ± 0.0118 | 0.1431 ± 0.0107 | 0.9513 |
| Return velocity (µm/s) | 3.378 ± 0.1763 | 3.142 ± 0.3837 | 0.4481 |


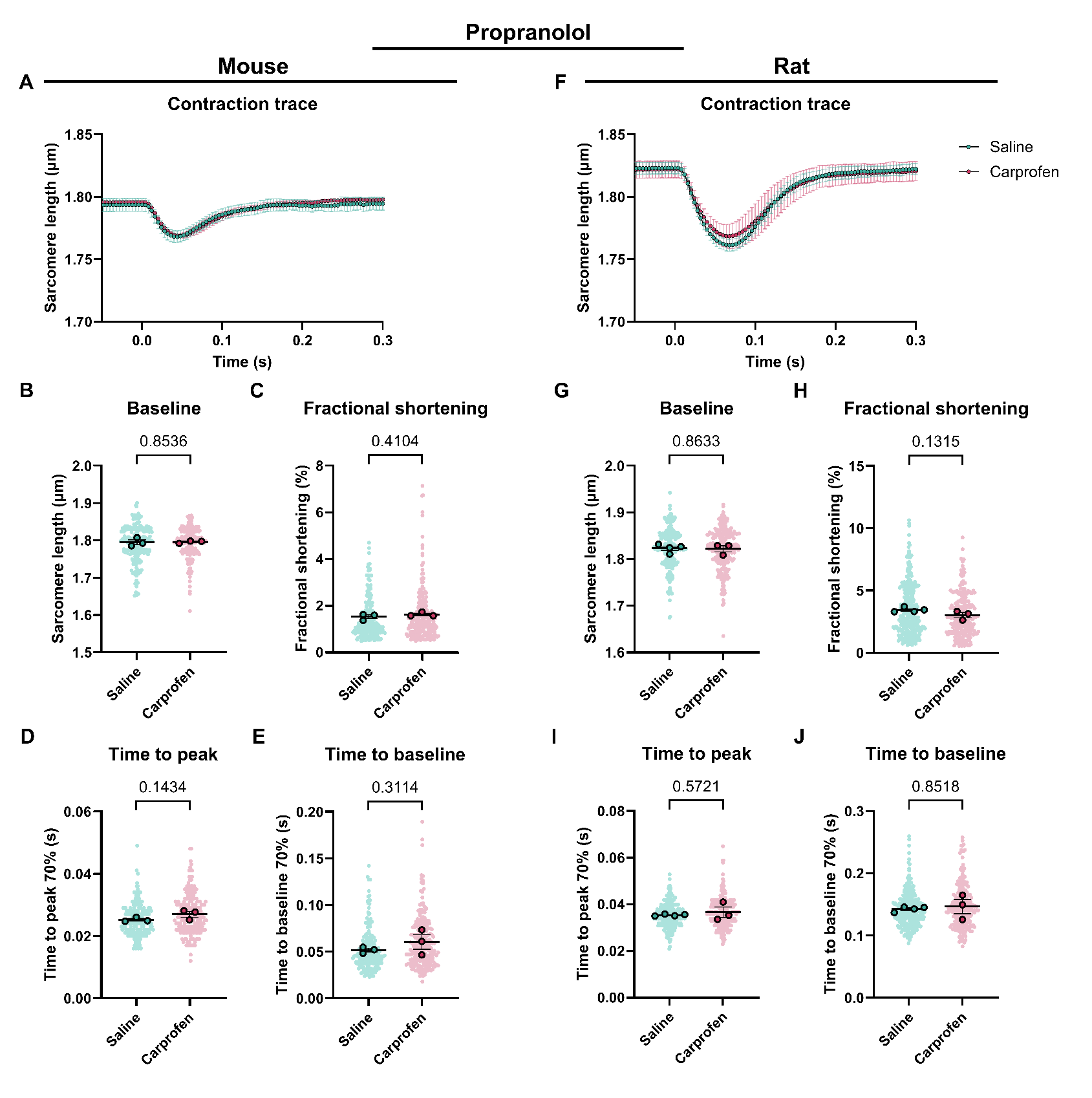


**Figure S3: Contractility of propranolol-treated cardiomyocytes derived from mice and rats systemically injected with saline or carprofen.** (A) Average contraction traces, (B) baseline sarcomere length, (C) fractional shortening, (D) time to peak and (E) time to baseline of cardiomyocytes treated with propranolol derived from mice injected with saline (N=3, n=206) or carprofen (N=3, n=236). (F) Average contraction traces, (G) baseline sarcomere length, (H) fractional shortening, (I) time to peak and (J) time to baseline of cardiomyocytes treated with propranolol derived from rats injected with saline (N=4, n=285) or carprofen (N=3, n=244). Data are expressed as mean ± standard error of the mean. Every small symbol represents the value of a single cardiomyocyte, n, and every bigger symbol with black outline represents the average per animal, N. Linear mixed model statistics was performed, taking the analgesia as fixed effect and the different animals as random effect. Data was transformed to correct for non-normality. No post-hoc test were performed. P-values are shown in the graphs. Data was considered significant with p < 0.05.


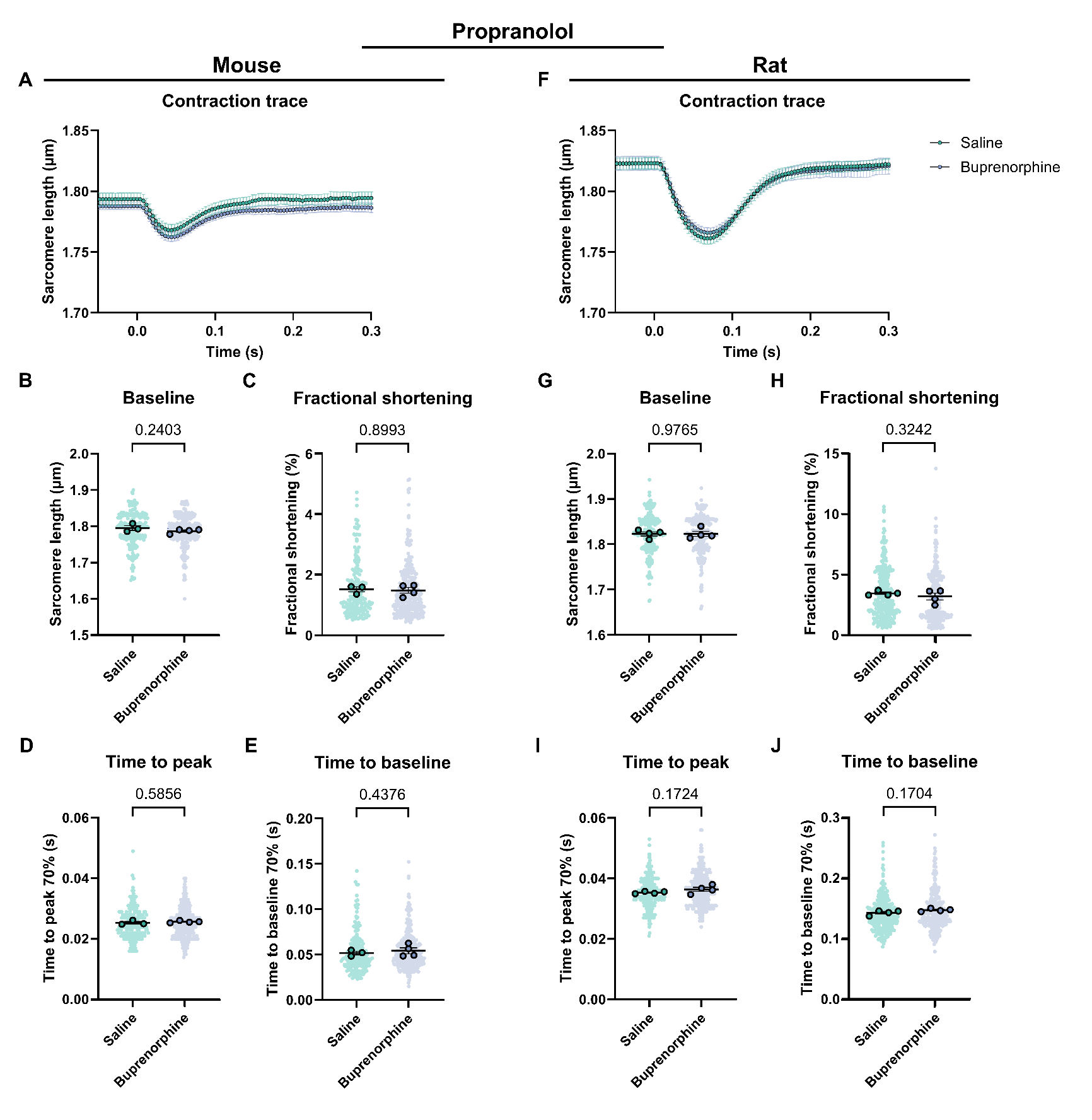


**Figure S4: Contractility of propranolol-treated cardiomyocytes derived from mice and rats systemically injected with saline or buprenorphine.** (A) Average contraction traces, (B) baseline sarcomere length, (C) fractional shortening, (D) time to peak and (E) time to baseline of cardiomyocytes treated with propranolol derived from mice injected with saline (N=3, n=206) or buprenorphine (N=4, n=315). (F) Average contraction traces, (G) baseline sarcomere length, (H) fractional shortening, (I) time to peak and (J) time to baseline of cardiomyocytes treated with propranolol derived from rats injected with saline (N=4, n=285) or buprenorphine (N=3, n=296). Data are expressed as mean ± standard error of the mean. Every small symbol represents the value of a single cardiomyocyte, n, and every bigger symbol with black outline represents the average per animal, N. Linear mixed model statistics was performed, taking the analgesia as fixed effect and the different animals as random effect. Data was transformed to correct for non-normality. No post-hoc test were performed. P-values are shown in the graphs. Data was considered significant with p < 0.05.

**Table S14: Contractility differences between propranolol-treated cardiomyocytes of mice and rats systemically injected with saline or carprofen.** Data are expressed as mean ± standard error of the mean. N represents the number of animals and n represents the number of single cardiomyocytes measured per condition. Linear mixed model statistics was performed, taking the analgesia as fixed effect and the different animals as random effect. Data was transformed to correct for non-normality. No post-hoc test were performed. P-values, N and n are shown in the table. Data was considered significant with p < 0.05. This table shows additional contractility parameters belonging to Figure S3.

| Mice | | | |
| --- | --- | --- | --- |
|  | Saline *(N=3, n=206)* | Carprofen *(N=3, n=236)* | *P* value |
| Baseline (µm) | 1.794 ± 0.0051 | 1.796 ± 0.0014 | 0.8536 |
| Fractional shortening (%) | 1.435 ± 0.0512 | 1.530 ± 0.0576 | 0.4104 |
| Time to peak 30% (s) | 0.015 ± 0.0004 | 0.016 ± 0.0004 | 0.0590 |
| Time to peak 50% (s) | 0.020 ± 0.0004 | 0.021 ± 0.0007 | **0.0459** |
| Time to peak 70% (s) | 0.025 ± 0.0004 | 0.027 ± 0.0011 | 0.1434 |
| Time to peak 90% (s) | 0.032 ± 0.0005 | 0.036 ± 0.0018 | 0.1749 |
| Departure velocity (µm/s) | 1.207 ± 0.0616 | 1.148 ± 0.0727 | 0.8367 |
| Time to baseline 30% (s) | 0.070 ± 0.0022 | 0.078 ± 0.0063 | 0.2466 |
| Time to baseline 50% (s) | 0.082 ± 0.0025 | 0.092 ± 0.0079 | 0.2667 |
| Time to baseline 70% (s) | 0.100 ± 0.0032 | 0.111 ± 0.0101 | 0.3114 |
| Time to baseline 90% (s) | 0.122 ± 0.0073 | 0.095 ± 0.0160 | 0.2632 |
| Return velocity (µm/s) | 0.599 ± 0.0675 | 0.843 ± 0.1197 | 0.4764 |
| Rats | | | |
|  | Saline *(N=4, n=285)* | Carprofen *(N=3, n=244)* | *P* value |
| Baseline (µm) | 1.823 ± 0.0047 | 1.822 ± 0.0068 | 0.8633 |
| Fractional shortening (%) | 3.181 ± 0.2974 | 2.992 ± 0.2127 | 0.1315 |
| Time to peak 30% (s) | 0.020 ± 0.0004 | 0.021 ± 0.0013 | 0.5509 |
| Time to peak 50% (s) | 0.026 ± 0.0005 | 0.027 ± 0.0016 | 0.5220 |
| Time to peak 70% (s) | 0.035 ± 0.0006 | 0.036 ± 0.0022 | 0.5721 |
| Time to peak 90% (s) | 0.049 ± 0.0005 | 0.050 ± 0.0031 | 0.7026 |
| Departure velocity (µm/s) | 1.846 ± 0.1370 | 1.658 ± 0.1181 | 0.1189 |
| Time to baseline 30% (s) | 0.109 ± 0.0031 | 0.107 ± 0.0084 | 0.9796 |
| Time to baseline 50% (s) | 0.125 ± 0.0044 | 0.122 ± 0.0094 | 0.9900 |
| Time to baseline 70% (s) | 0.143 ± 0.0057 | 0.142 ± 0.0109 | 0.8518 |
| Time to baseline 90% (s) | 0.127 ± 0.0168 | 0.113 ± 0.0228 | 0.5742 |
| Return velocity (µm/s) | 1.795 ± 0.6115 | 1.678 ± 0.7194 | 0.1963 |

**Table S15: Contractility differences between propranolol-treated cardiomyocytes of mice and rats systemically injected with saline or buprenorphine.** Data are expressed as mean ± standard error of the mean. N represents the number of animals and n represents the number of single cardiomyocytes measured per condition. Linear mixed model statistics was performed, taking the analgesia as fixed effect and the different animals as random effect. Data was transformed to correct for non-normality. No post-hoc test were performed. P-values, N and n are shown in the table. Data was considered significant with p < 0.05. This table shows additional contractility parameters belonging to Figure S4.

| Mice | | | |
| --- | --- | --- | --- |
|  | Saline *(N=3, n=206)* | Buprenorphine *(N=4, n=315)* | *P* value |
| Baseline (µm) | 1.794 ± 0.0051 | 1.788 ± 0.0028 | 0.2403 |
| Fractional shortening (%) | 1.435 ± 0.0512 | 1.432 ± 0.0803 | 0.8993 |
| Time to peak 30% (s) | 0.015 ± 0.0004 | 0.019 ± 0.0001 | 0.8809 |
| Time to peak 50% (s) | 0.020 ± 0.0004 | 0.019 ± 0.0002 | 0.7324 |
| Time to peak 70% (s) | 0.025 ± 0.0004 | 0.025 ± 0.0002 | 0.5856 |
| Time to peak 90% (s) | 0.032 ± 0.0005 | 0.033 ± 0.0004 | 0.3678 |
| Departure velocity (µm/s) | 1.207 ± 0.0616 | 1.146 ± 0.0728 | 0.8633 |
| Time to baseline 30% (s) | 0.070 ± 0.0022 | 0.072 ± 0.0017 | 0.3692 |
| Time to baseline 50% (s) | 0.082 ± 0.0025 | 0.085 ± 0.0023 | 0.4010 |
| Time to baseline 70% (s) | 0.100 ± 0.0032 | 0.105 ± 0.0029 | 0.4376 |
| Time to baseline 90% (s) | 0.122 ± 0.0073 | 0.121 ± 0.0131 | 0.4650 |
| Return velocity (µm/s) | 0.599 ± 0.0675 | 0.550 ± 0.1057 | 0.7316 |
| Rats | | | |
|  | Saline *(N=4, n=285)* | Buprenorphine *(N=4, n=296)* | *P* value |
| Baseline (µm) | 1.823 ± 0.0047 | 1.823 ± 0.0058 | 0.9765 |
| Fractional shortening (%) | 3.181 ± 0.2974 | 3.149 ± 0.2790 | 0.3242 |
| Time to peak 30% (s) | 0.020 ± 0.0004 | 0.021 ± 0.0004 | 0.3095 |
| Time to peak 50% (s) | 0.026 ± 0.0005 | 0.028 ± 0.0004 | 0.0808 |
| Time to peak 70% (s) | 0.035 ± 0.0006 | 0.036 ± 0.0006 | 0.1724 |
| Time to peak 90% (s) | 0.049 ± 0.0005 | 0.050 ± 0.0011 | 0.3859 |
| Departure velocity (µm/s) | 1.846 ± 0.1370 | 1.747 ± 0.1245 | 0.1276 |
| Time to baseline 30% (s) | 0.109 ± 0.0031 | 0.108 ± 0.0020 | 0.3288 |
| Time to baseline 50% (s) | 0.125 ± 0.0044 | 0.124 ± 0.0019 | 0.2887 |
| Time to baseline 70% (s) | 0.143 ± 0.0057 | 0.143 ± 0.0020 | 0.1704 |
| Time to baseline 90% (s) | 0.127 ± 0.0168 | 0.109 ± 0.0232 | 0.3075 |
| Return velocity (µm/s) | 1.795 ± 0.6115 | 1.551 ± 0.1822 | 0.3340 |

**Table S16: Contractility differences between acutely saline- or carprofen-treated cardiomyocytes in combination with no or isoprenaline treatment of rats systemically injected with saline.** Data are expressed as mean ± standard error of the mean. N represents the number of animals and n represents the number of single cardiomyocytes measured per condition. Linear mixed model statistics was performed, taking the analgesia as fixed effect and the different animals as random effect. Data was transformed to correct for non-normality. No post-hoc test were performed. P-values, N and n are shown in the table. Data was considered significant with p < 0.05. This table shows additional contractility parameters belonging to Figure 7.

| Control | | | |
| --- | --- | --- | --- |
|  | Saline *(N=3, n=197)* | Carprofen *(N=3, n=214)* | *P* value |
| Baseline (µm) | 1.784 ± 0.0075 | 1.754 ± 0.0099 | **0.0450** |
| Fractional shortening (%) | 4.837 ± 0.2574 | 4.979 ± 0.2314 | 0.5830 |
| Time to peak 30% (s) | 0.018 ± 0.0005 | 0.019 ± 0.0006 | 0.5155 |
| Time to peak 50% (s) | 0.025 ± 0.0007 | 0.025 ± 0.0009 | 0.5756 |
| Time to peak 70% (s) | 0.033 ± 0.0008 | 0.034 ± 0.0012 | 0.4507 |
| Time to peak 90% (s) | 0.047 ± 0.0011 | 0.049 ± 0.0020 | 0.8186 |
| Departure velocity (µm/s) | 2.886 ± 0.1911 | 2.759 ± 0.2424 | 0.7325 |
| Time to baseline 30% (s) | 0.100 ± 0.0032 | 0.106 ± 0.0054 | 0.2954 |
| Time to baseline 50% (s) | 0.113 ± 0.0040 | 0.120 ± 0.0059 | 0.3674 |
| Time to baseline 70% (s) | 0.127 ± 0.0049 | 0.137 ± 0.0059 | 0.4550 |
| Time to baseline 90% (s) | 0.145 ± 0.0096 | 0.129 ± 0.0057 | 0.2087 |
| Return velocity (µm/s) | 3.692 ± 1.9266 | 4.566 ± 2.9331 | 0.9484 |
| Isoprenaline | | | |
|  | Saline *(N=3, n=210)* | Carprofen *(N=3, n=171)* | *P* value |
| Baseline (µm) | 1.739 ± 0.0075 | 1.696 ± 0.0052 | **0.0025** |
| Fractional shortening (%) | 6.664 ± 0.2756 | 8.231 ± 1.2677 | 0.2872 |
| Time to peak 30% (s) | 0.017 ± 0.0007 | 0.017 ± 0.0010 | 0.8451 |
| Time to peak 50% (s) | 0.023 ± 0.0006 | 0.023 ± 0.0016 | 0.8490 |
| Time to peak 70% (s) | 0.032 ± 0.0007 | 0.031 ± 0.0022 | 0.7606 |
| Time to peak 90% (s) | 0.046 ± 0.0006 | 0.046 ± 0.0021 | 0.9588 |
| Departure velocity (µm/s) | 4.013 ± 0.2153 | 5.080 ± 1.0906 | 0.4766 |
| Time to baseline 30% (s) | 0.101 ± 0.0015 | 0.099 ± 0.0048 | 0.8676 |
| Time to baseline 50% (s) | 0.112 ± 0.0016 | 0.109 ± 0.0052 | 0.7535 |
| Time to baseline 70% (s) | 0.123 ± 0.0021 | 0.121 ± 0.0065 | 0.3675 |
| Time to baseline 90% (s) | 0.148 ± 0.0018 | 0.147 ± 0.0101 | 0.4957 |
| Return velocity (µm/s) | 3.024 ± 0.4524 | 4.928 ± 0.8721 | 0.2926 |

**Table S17: Contractility differences between acutely saline- or buprenorphine-treated cardiomyocytes in combination with no or isoprenaline treatment of rats systemically injected with saline.** Data are expressed as mean ± standard error of the mean. N represents the number of animals and n represents the number of single cardiomyocytes measured per condition. Linear mixed model statistics was performed, taking the analgesia as fixed effect and the different animals as random effect. Data was transformed to correct for non-normality. No post-hoc test were performed. P-values, N and n are shown in the table. Data was considered significant with p < 0.05. This table shows additional contractility parameters belonging to Figure 8.

| Control | | | |
| --- | --- | --- | --- |
|  | Saline *(N=3, n=197)* | Buprenorphine *(N=3, n=211)* | *P* value |
| Baseline (µm) | 1.784 ± 0.0075 | 1.776 ± 0.0071 | 0.7176 |
| Fractional shortening (%) | 4.837 ± 0.2574 | 4.903 ± 0.3010 | 0.9102 |
| Time to peak 30% (s) | 0.018 ± 0.0005 | 0.019 ± 0.0010 | 0.0922 |
| Time to peak 50% (s) | 0.025 ± 0.0007 | 0.025 ± 0.0012 | 0.7778 |
| Time to peak 70% (s) | 0.033 ± 0.0008 | 0.034 ± 0.0015 | 0.7195 |
| Time to peak 90% (s) | 0.047 ± 0.0011 | 0.048 ± 0.0019 | 0.6796 |
| Departure velocity (µm/s) | 2.886 ± 0.1911 | 2.841 ± 0.3072 | 0.8989 |
| Time to baseline 30% (s) | 0.100 ± 0.0032 | 0.104 ± 0.0054 | 0.6321 |
| Time to baseline 50% (s) | 0.113 ± 0.0040 | 0.117 ± 0.0064 | 0.6848 |
| Time to baseline 70% (s) | 0.127 ± 0.0049 | 0.131 ± 0.0071 | 0.8198 |
| Time to baseline 90% (s) | 0.145 ± 0.0096 | 0.136 ± 0.0063 | 0.5896 |
| Return velocity (µm/s) | 3.692 ± 1.9266 | 2.758 ± 0.7460 | 0.9896 |
| Isoprenaline | | | |
|  | Saline *(N=3, n=210)* | Buprenorphine *(N=3, n=211)* | *P* value |
| Baseline (µm) | 1.739 ± 0.0075 | 1.731 ± 0.0084 | 0.5331 |
| Fractional shortening (%) | 6.664 ± 0.2756 | 8.385 ± 0.5301 | **0.0153** |
| Time to peak 30% (s) | 0.017 ± 0.0007 | 0.016 ± 0.0005 | 0.2074 |
| Time to peak 50% (s) | 0.023 ± 0.0006 | 0.022 ± 0.0007 | 0.1852 |
| Time to peak 70% (s) | 0.032 ± 0.0007 | 0.030 ± 0.0009 | 0.1697 |
| Time to peak 90% (s) | 0.046 ± 0.0006 | 0.044 ± 0.0016 | 0.2626 |
| Departure velocity (µm/s) | 4.013 ± 0.2153 | 5.383 ± 0.5274 | **0.0373** |
| Time to baseline 30% (s) | 0.101 ± 0.0015 | 0.095 ± 0.0059 | 0.4059 |
| Time to baseline 50% (s) | 0.112 ± 0.0016 | 0.106 ± 0.0073 | 0.3722 |
| Time to baseline 70% (s) | 0.123 ± 0.0021 | 0.117 ± 0.0090 | 0.3819 |
| Time to baseline 90% (s) | 0.148 ± 0.0018 | 0.141 ± 0.0115 | 0.3063 |
| Return velocity (µm/s) | 3.024 ± 0.4524 | 6.707 ± 1.9488 | 0.0988 |


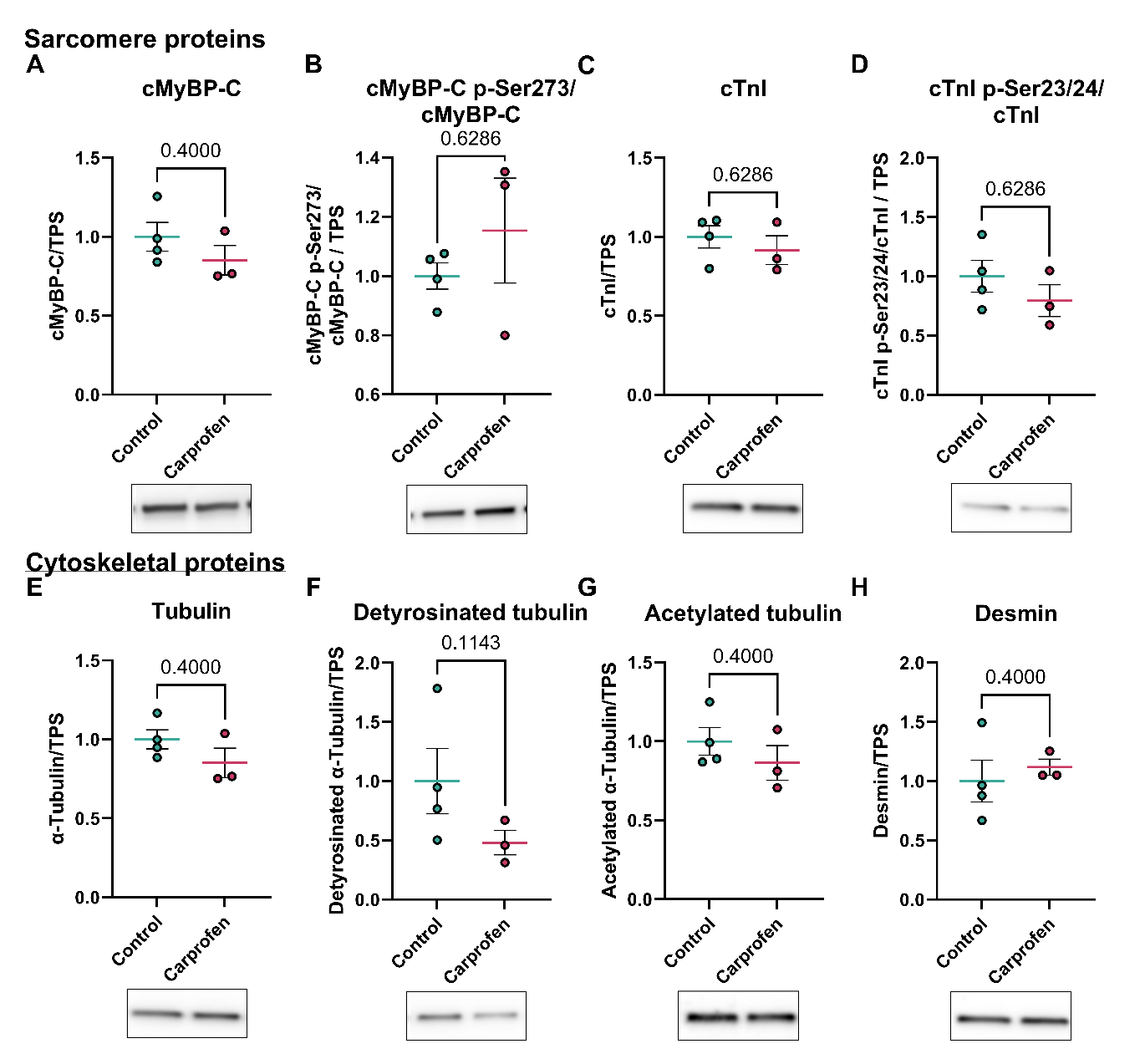


**Figure S5: Protein expression of hearts derived from rats systemically injected with saline or carprofen.** Quantification of the protein expression of sarcomere proteins (A) cMyBP-C, (B) cMyBP-C p-Ser273/cMyBP-C, (C) cTnI and (D) cTnI p-Ser 23/24/cTnI and cytoskeletal proteins (E) α-tubulin, (F) detyrosinated α-tubulin, (G) acetylated α-tubulin and (H) desmin of rat hearts injected with saline (N=4) or carprofen (N=3). Protein expression was normalized to the total protein stain. Data are expressed as mean ± standard error of the mean. Each sample was blotted in duplo. Every symbol represents the average per animal, N. Mann-Whitney tests were performed. No post-hoc test were performed. P-values are shown in the graphs. Data was considered significant with p < 0.05.


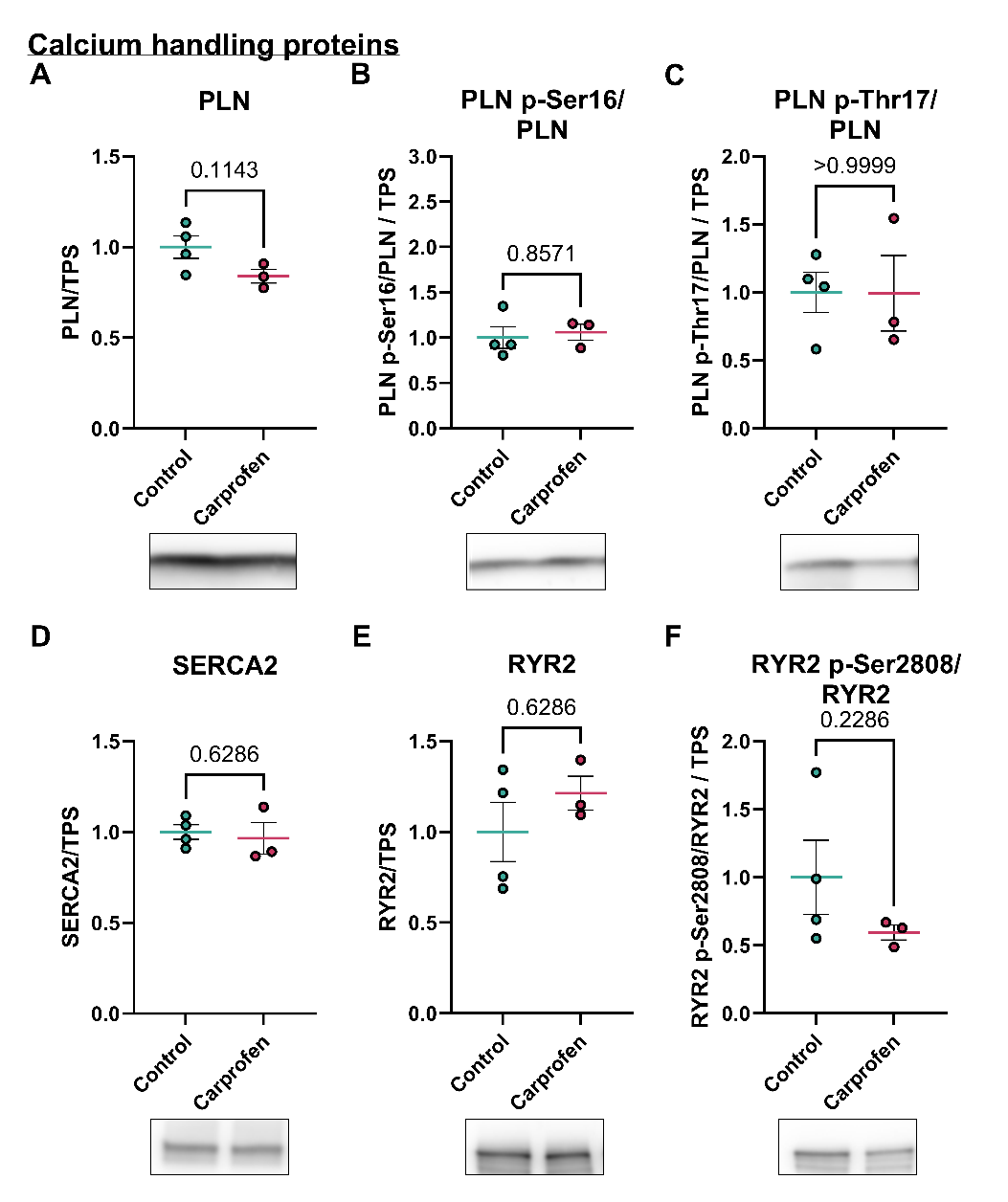


**Figure S6: Protein expression of hearts derived from rats systemically injected with saline or carprofen.** Quantification of the protein expression of calcium handling proteins (A) PLN, (B) PLN p-Ser16/PLN, (C) PLN p-Thr17/PLN, (D) SERCA2, (E) RYR2 and (F) RYR2 p-Ser2808/RYR2 of rat hearts injected with saline (N=4) or carprofen (N=3). Protein expression was normalized to the total protein stain. Data are expressed as mean ± standard error of the mean. Each sample was blotted in duplo. Every symbol represents the average per animal, N. Mann-Whitney tests were performed. No post-hoc test were performed. P-values are shown in the graphs. Data was considered significant with p < 0.05.


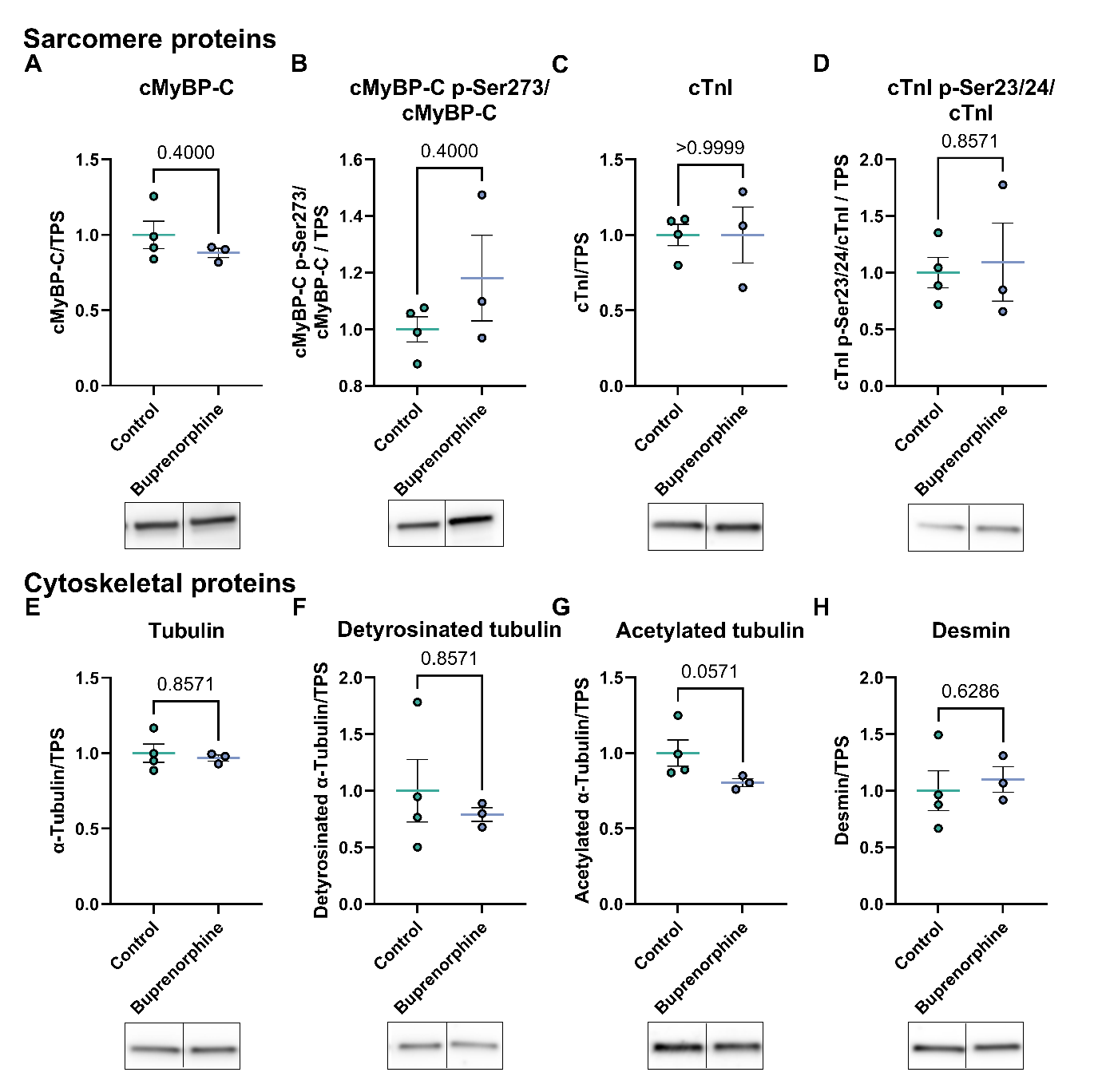

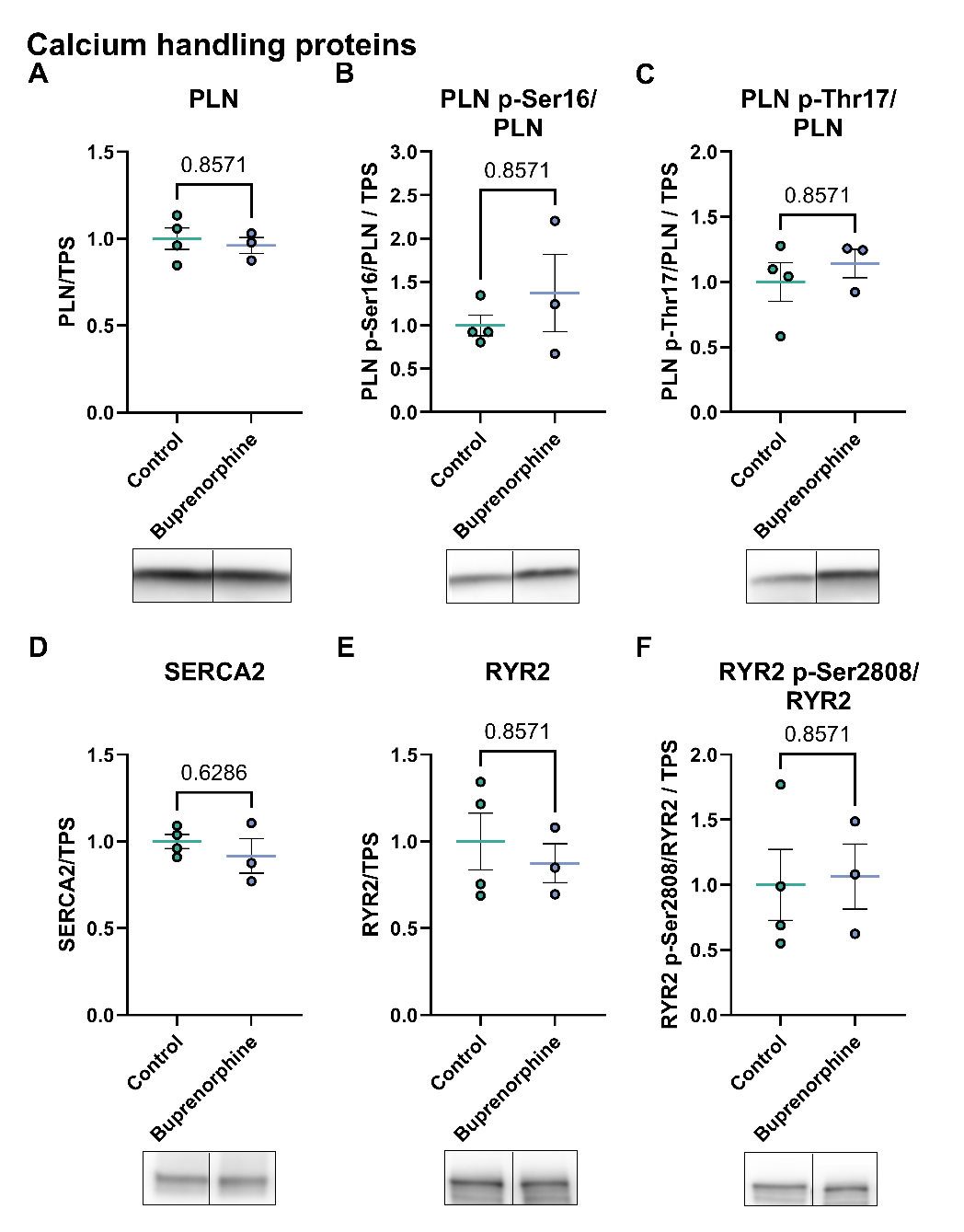


**Figure S7: Protein expression of hearts derived from rats systemically injected with saline or buprenorphine.** Quantification of the protein expression of sarcomere proteins (A) cMyBP-C, (B) cMyBP-C p-Ser273/cMyBP-C, (C) cTnI and (D) cTnI p-Ser 23/24/cTnI and cytoskeletal proteins (E) α-tubulin, (F) detyrosinated α-tubulin, (G) acetylated α-tubulin and (H) desmin of rat hearts injected with saline (N=4) or buprenorphine (N=3). Protein expression was normalized to the total protein stain. Data are expressed as mean ± standard error of the mean. Each sample was blotted in duplo. Every symbol represents the average per animal, N. Mann-Whitney tests were performed. No post-hoc test were performed. P-values are shown in the graphs. Data was considered significant with p < 0.05.

**Figure S8: Protein expression of hearts derived from rats systemically injected with saline or buprenorphine.** Quantification of the protein expression of calcium handling proteins (A) PLN, (B) PLN p-Ser16/PLN, (C) PLN p-Thr17/PLN, (D) SERCA2, (E) RYR2 and (F) RYR2 p-Ser2808/RYR2 of rat hearts injected with saline (N=4) or buprenorphine (N=3). Protein expression was normalized to the total protein stain. Data are expressed as mean ± standard error of the mean. Each sample was blotted in duplo. Every symbol represents the average per animal, N. Mann-Whitney tests were performed. No post-hoc test were performed. P-values are shown in the graphs. Data was considered significant with p < 0.05.

**Figure S9: Images of the western blots of hearts derived from rats injected with saline, carprofen or buprenorphine.**


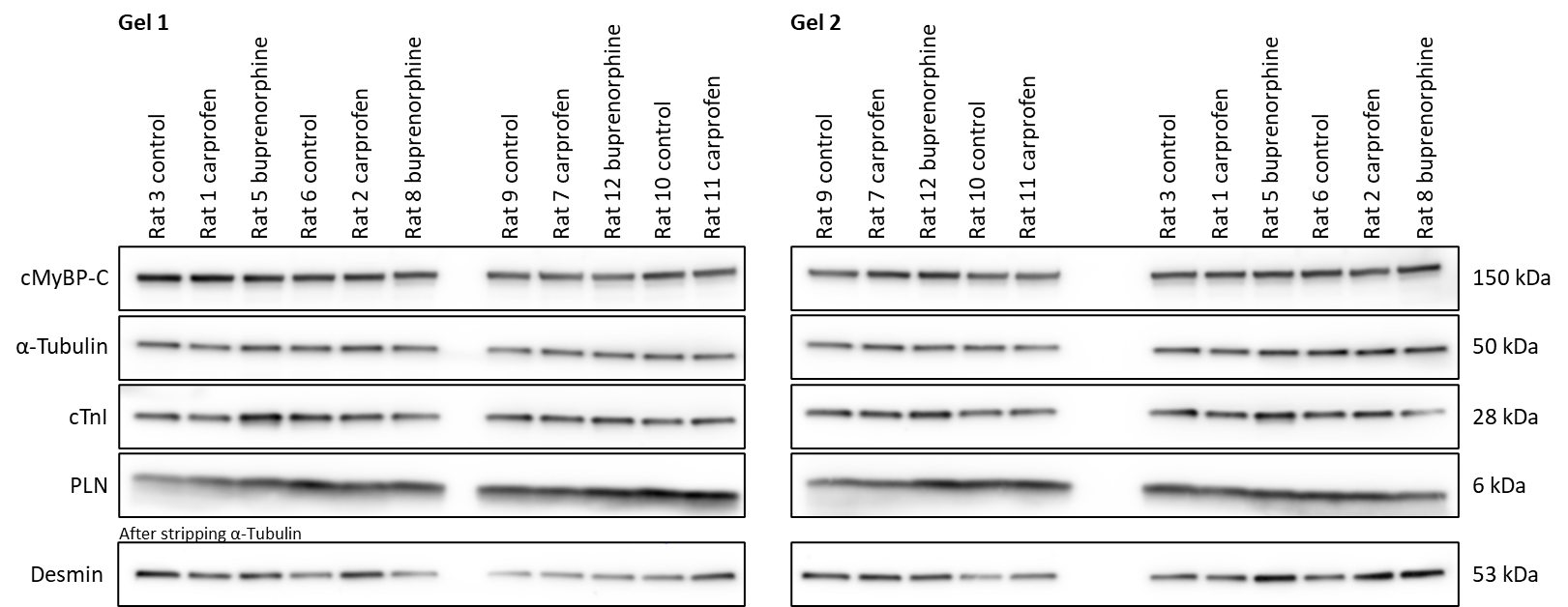

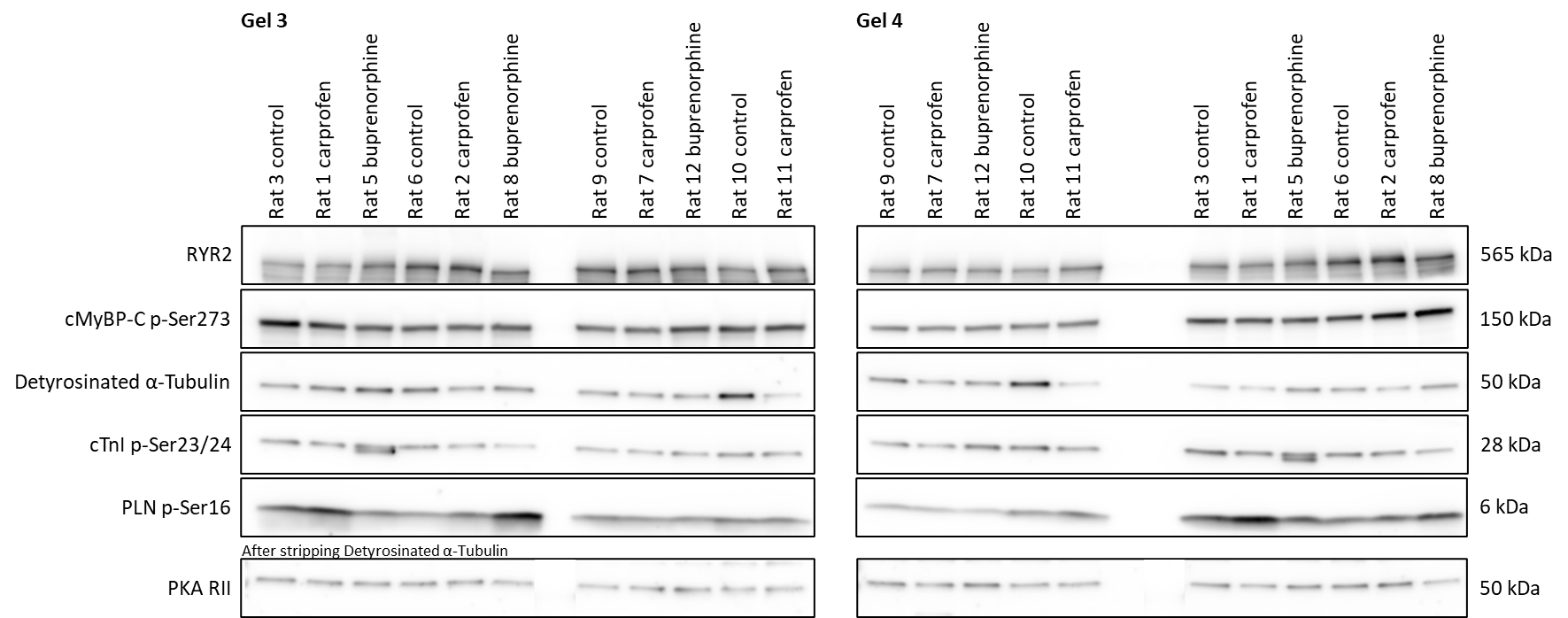

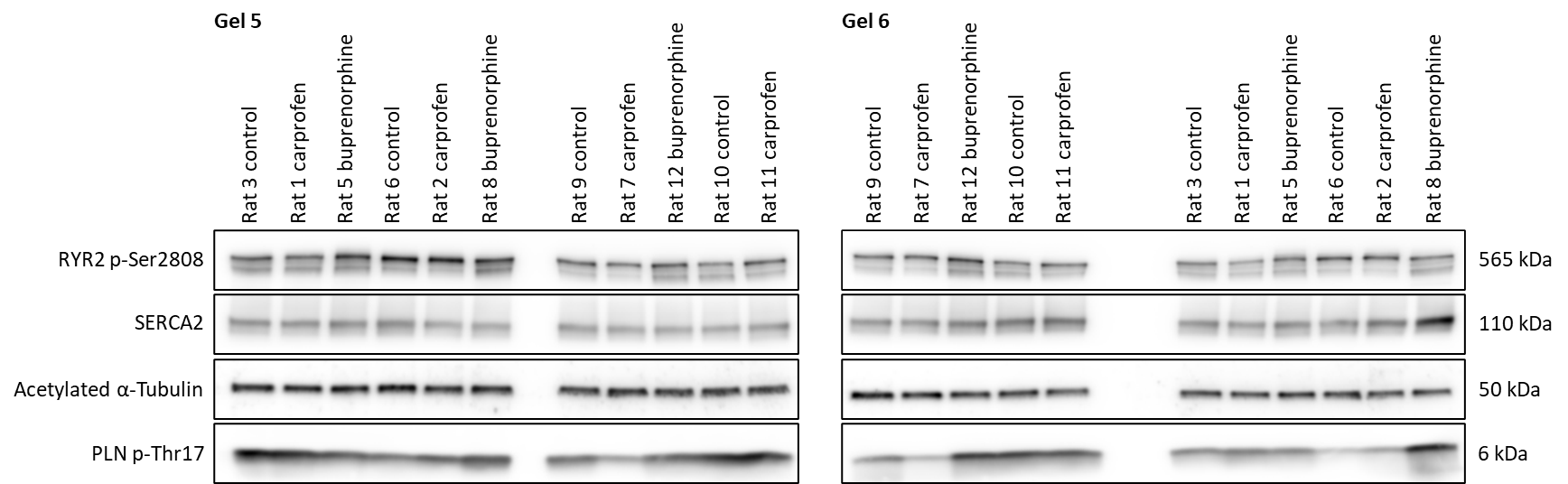

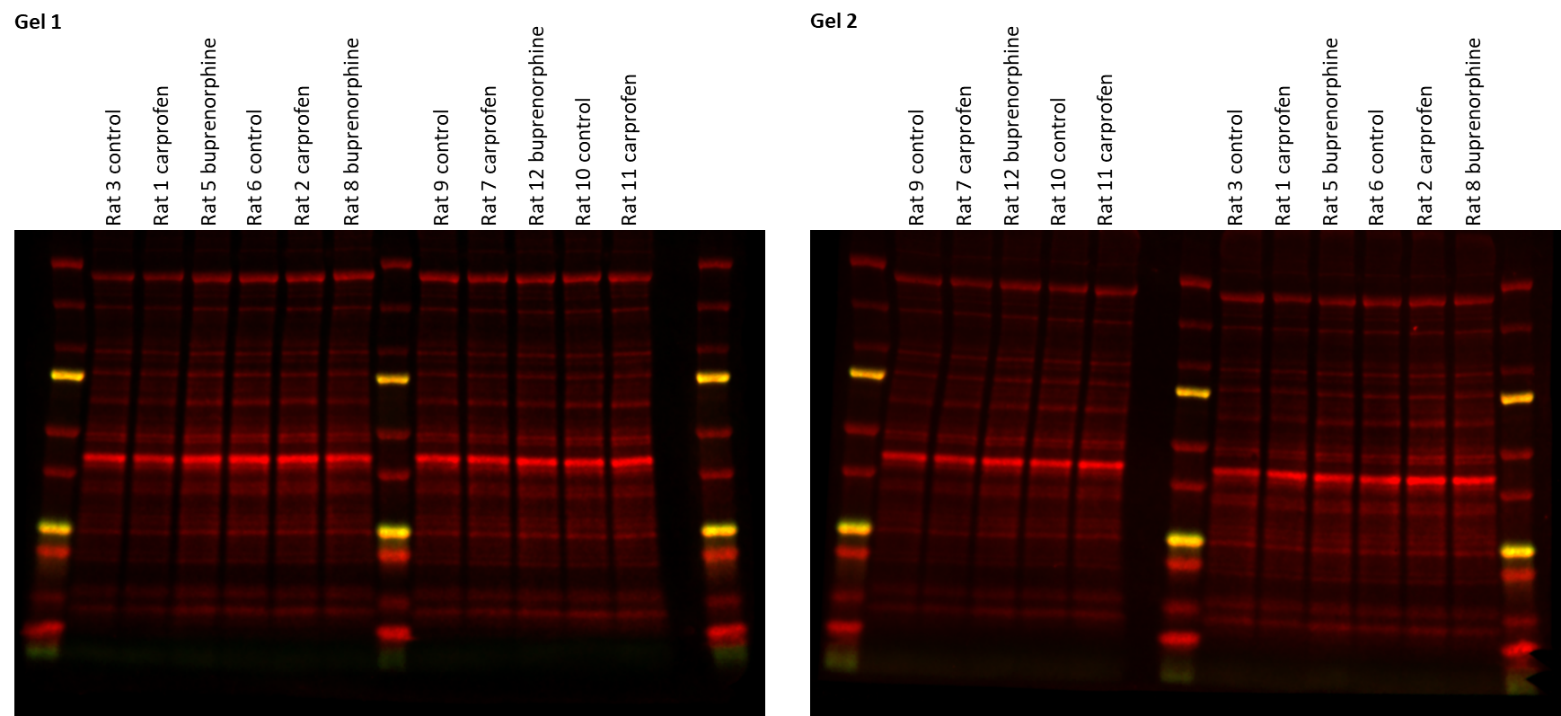

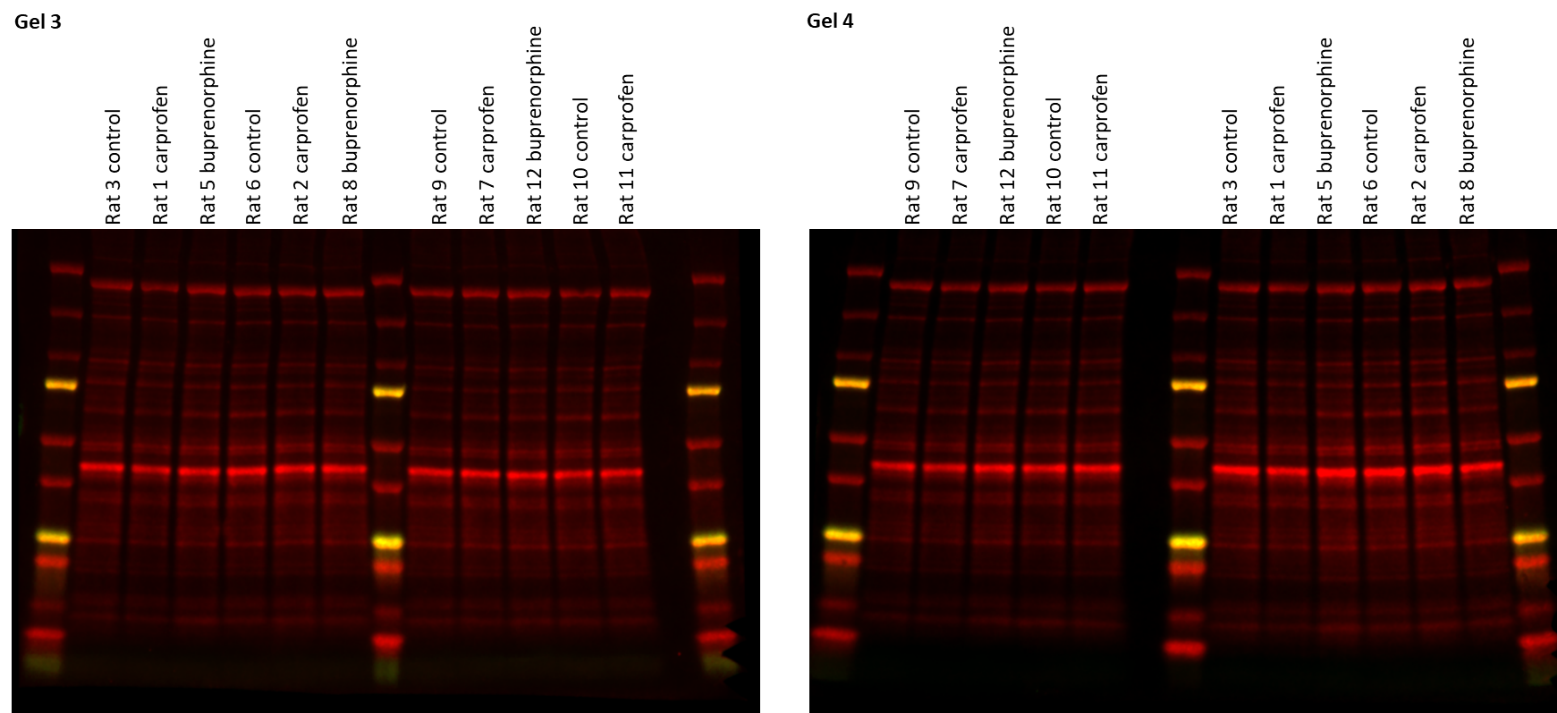

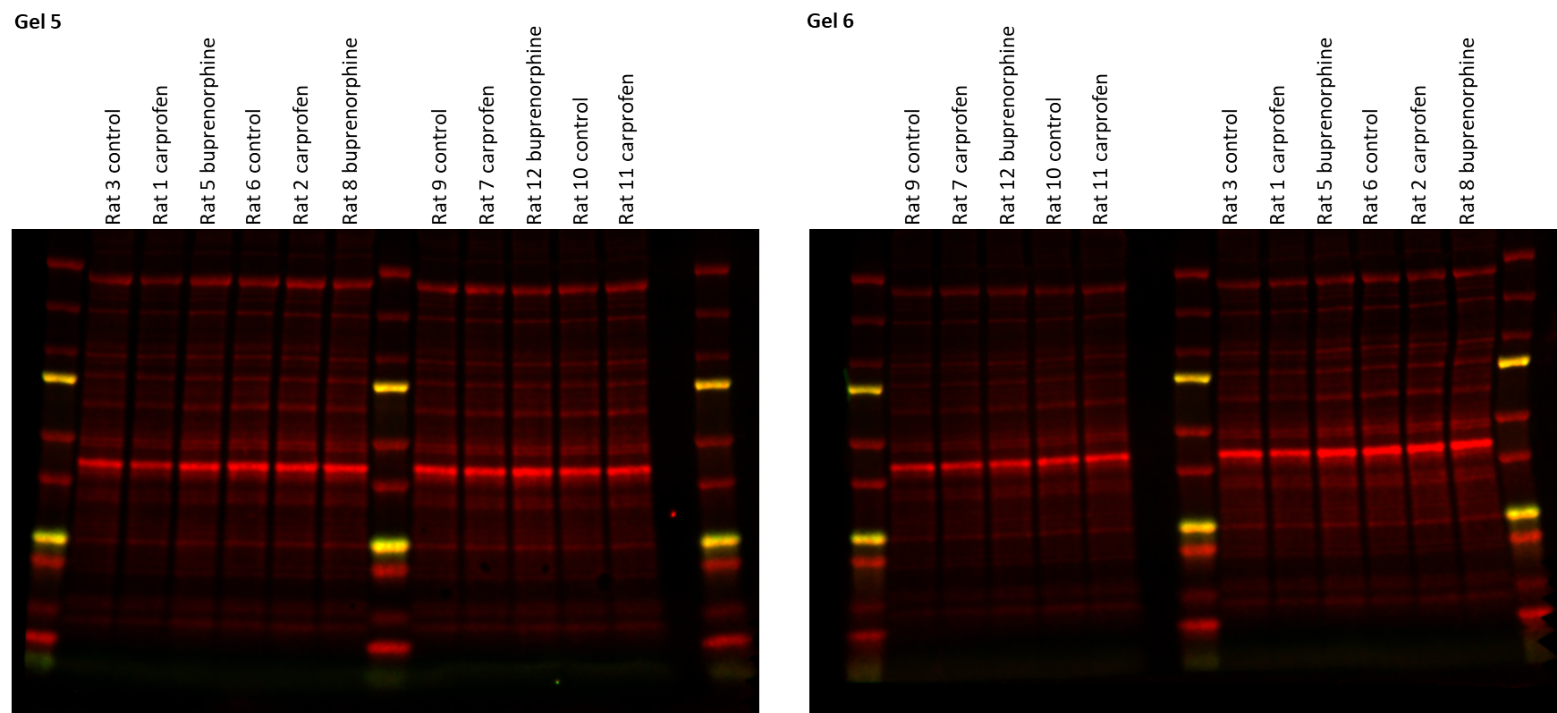


**Figure S10: Images of the total protein stains of hearts derived from rats injected with saline, carprofen or buprenorphine.**
